# Controls of Nucleosome Positioning in the Human Genome

**DOI:** 10.1371/journal.pgen.1003036

**Published:** 2012-11-15

**Authors:** Daniel J. Gaffney, Graham McVicker, Athma A. Pai, Yvonne N. Fondufe-Mittendorf, Noah Lewellen, Katelyn Michelini, Jonathan Widom, Yoav Gilad, Jonathan K. Pritchard

**Affiliations:** 1 Department of Human Genetics, University of Chicago, Chicago, Illinois, United States of America; 2 Howard Hughes Medical Institute, Chevy Chase, Maryland, United States of America; 3 Wellcome Trust Sanger Institute, Hinxton, United Kingdom; 4 Department of Molecular Biosciences and Department of Chemistry, Northwestern University, Chicago, Illinois, United States of America; 5 Department of Molecular and Cellular Biochemistry, University of Kentucky, Lexington, Kentucky, United States of America; Weizmann Institute of Science, Israel

## Abstract

Nucleosomes are important for gene regulation because their arrangement on the genome can control which proteins bind to DNA. Currently, few human nucleosomes are thought to be consistently positioned across cells; however, this has been difficult to assess due to the limited resolution of existing data. We performed paired-end sequencing of micrococcal nuclease-digested chromatin (MNase–seq) from seven lymphoblastoid cell lines and mapped over 3.6 billion MNase–seq fragments to the human genome to create the highest-resolution map of nucleosome occupancy to date in a human cell type. In contrast to previous results, we find that most nucleosomes have more consistent positioning than expected by chance and a substantial fraction (8.7%) of nucleosomes have moderate to strong positioning. In aggregate, nucleosome sequences have 10 bp periodic patterns in dinucleotide frequency and DNase I sensitivity; and, across cells, nucleosomes frequently have translational offsets that are multiples of 10 bp. We estimate that almost half of the genome contains regularly spaced arrays of nucleosomes, which are enriched in active chromatin domains. Single nucleotide polymorphisms that reduce DNase I sensitivity can disrupt the phasing of nucleosome arrays, which indicates that they often result from positioning against a barrier formed by other proteins. However, nucleosome arrays can also be created by DNA sequence alone. The most striking example is an array of over 400 nucleosomes on chromosome 12 that is created by tandem repetition of sequences with strong positioning properties. In summary, a large fraction of nucleosomes are consistently positioned—in some regions because they adopt favored sequence positions, and in other regions because they are forced into specific arrangements by chromatin remodeling or DNA binding proteins.

## Author Summary

Within the nucleus of the cell, the genome of eukaryotic organisms is tightly packaged into chromatin. Chromatin is composed of a repeating series of bead-like nucleosomes, each of which is encircled 1.7 times by a string of DNA. The organization of nucleosomes on the genome is fundamentally important because they can prevent other proteins from accessing the DNA. Previous studies of human nucleosomes concluded that most nucleosomes have fuzzy positioning and tend to occupy different locations in different cells. This interpretation, however, may be a consequence of the low resolution of existing data. Here we revisit the question of nucleosome positioning by generating the most precise map of nucleosome positions that has ever been created for a human cell line. We find that 8.7% of nucleosomes have very consistent positioning, and most nucleosomes are more consistently positioned than expected by chance. Additionally, we estimate that almost half of the genome contains regularly spaced arrays of nucleosomes. Much of this positioning is due to the intrinsic preference of nucleosomes for some DNA sequences over others; but in some regions of the genome, the sequence preferences of nucleosomes are overridden by proteins that out-compete them for binding or displace them using energy from ATP.

## Introduction

In eukaryotes, the genome is organized into a compact protein-DNA complex known as chromatin which, at the most fundamental level, consists of a repeating series of nucleosome core “beads” separated by linker DNA “strings” [1]. Each nucleosome core is made up of an octamer of histone proteins, encircled 1.7 times by 147 base pairs of DNA [2]. Since nucleosomes sterically exclude other proteins that bind to DNA, their organization on the genome sequence can dictate which sites are accessible to regulatory factors and transcriptional machinery [3], [4].

Nucleosome organization is described by the translational and rotational positions of nucleosomes [5]. The “translational” position is the stretch of DNA sequence that is wrapped around the nucleosome and can be specified by the genomic location of the nucleosome center. The “rotational” position is the orientation of the DNA helix as it wraps around the nucleosome. Since a single turn of DNA occupies about 10.5 bp, translational positions that are multiples of about 10.5 bp apart have similar rotational positions. Nucleosomes that adopt the same translational position in every cell are said to be “well” or “strongly” positioned and, conversely, those with highly variable positions are considered “weakly” or “fuzzily” positioned [5], [6].

The positioning of nucleosomes is at least partly encoded by the genome, because some DNA sequences energetically favor nucleosome formation more than others [7], [8]. Sequences such as Poly(dA:dT) tracts strongly disfavor nucleosome formation [9]–[12], and GC rich sequences tend to have high nucleosome occupancy [13], [14]. Periodic AA/AT/TA/TT dinucleotides may be particularly amenable to nucleosome formation because they possess an intrinsic curvature that facilitates wrapping around the histone octamer [15]. These dinucleotides also influence the rotational positioning of nucleosomes—AA/AT/TA/TT dinucleotides are favored where the DNA minor groove faces inwards towards the histone surface, and CC/CG/GC/GG dinucleotides are favored where the minor groove faces outwards [5], [15], [16]. In aggregate, these dinucleotides show clear periodic frequencies in nucleosomal sequences obtained *in vitro* [7], [16], [17], and *in vivo* from yeast, fly and chicken [5], [15], [16], [18].

Nucleosome organization is also influenced by barriers that exclude nucleosomes from stretches of sequence [19]. For example, barriers can be created by proteins that compete for binding to a sequence [20], or by ATP-dependent chromatin remodelers that actively displace nucleosomes [21]. Additionally, barriers can be created by other strongly-positioned nucleosomes or by sequences that exclude nucleosomes such as poly(dA:dT) tracts [14], [19]. If the density of nucleosomes around a barrier is high, they will form “statistically positioned” arrays with consistent phasing across cells. This occurs because the nucleosome adjacent to the barrier can only occupy positions that do not overlap the barrier. Subsequent nucleosomes are similarly constrained because the second nucleosome cannot overlap the first, the third cannot overlap the second, and so on [22]. Positioned arrays of nucleosomes have been observed around active promoters [11], [14], [18], [23]–[25], around binding sites for the insulator protein CTCF [14], [26], and around binding sites for the repressor protein NRSF [14].

The importance of sequence preferences for nucleosome positioning is controversial, and some authors have argued that the nucleosomes with the strongest positioning are usually directed by cellular machinery such as RNA polymerase, chromatin remodelers, or transcription factors [14], [24], [27]. In the human genome the contributions of sequence preferences and other cellular factors have been difficult to asses because of the limited resolution of existing maps of nucleosome occupancy, which have been generated using high-throughput single-end sequencing of micrococcal nuclease digested chromatin (MNase-seq) [14], [23]. Since the length of MNase digestion fragments varies substantially, the positions of nucleosomes inferred from single-end reads are imprecise. As a consequence, it is not known what fraction of the human genome contains positioned arrays of nucleosomes, and it is difficult to assess the consistency of fine-scale translational or rotational nucleosome positions from the data that are available. The most extensive study of human nucleosome positioning to date reported that a small proportion (20%) of nucleosomes have even weak detectable positioning [14], but this may reflect the limited resolution of the existing data.

To overcome these limitations and assess the strength of nucleosome positioning in the human genome, we performed paired-end MNase-seq on seven human lymphoblastoid cell lines (LCLs) derived from HapMap individuals. LCLs are an ideal model system for this problem because they have been extensively characterized by ENCODE [28], and our lab recently generated high-resolution maps of DNase I sensitivity for them [29], [30]. The HapMap individuals that the LCLs are derived from are almost completely genotyped by the 1000 Genomes Project [31], and we use this information to investigate the impact of genetic variation on nucleosome organization. Our study is the first to employ paired-end MNase-seq in a human model system, and we determine nucleosome positions with much greater precision than previously possible. Our results reveal a surprising consistency in nucleosome positions and illuminate a trade-off between sequence preferences and barriers created by bound transcription factors.

## Results

To determine the genomic positions of nucleosomes in seven human lymphoblastoid cell lines (LCLs), we combined micrococcal nuclease (MNase) digestion of chromatin with high-throughput paired-end and single-end DNA sequencing (MNase-seq). MNase preferentially cuts within linker DNA [32], so the size-selected fragments indicate the positions of nucleosomes when they are sequenced and mapped back to the genome [33]. We aligned the paired-end MNase-seq reads to the human genome and calculated the size of each DNA fragment from the separation of the paired reads (mean = 152 bp, SD = 11.5; Figure S1). We also mapped a set of single-end reads, for which we assumed a fragment size of 151 bp (corresponding to the median from the paired-end sequencing). As a quality filter, we discarded fragments of extreme size (outside the central 95% range of 126–184 bp) or poor mapping quality (Q<10). In total, 2.5 billion paired-end and 1.1 billion single-end fragments were retained (Table S1), which corresponds to approximately 240 fragments per nucleosome.

### Rotational positioning of human nucleosomes

To study the rotational and fine-scale translational positioning of human nucleosome sequences, we restricted ourselves to 130 million fragments of length 147 bp. Fragments that are substantially longer or shorter than this may result from over- or under-digestion of nucleosomal DNA and provide less precise estimates of individual nucleosome positions. A major advantage of using paired-end sequencing is that large and small fragments can be filtered and the location of nucleosome dyads can be determined much more precisely.

**Figure 1:**
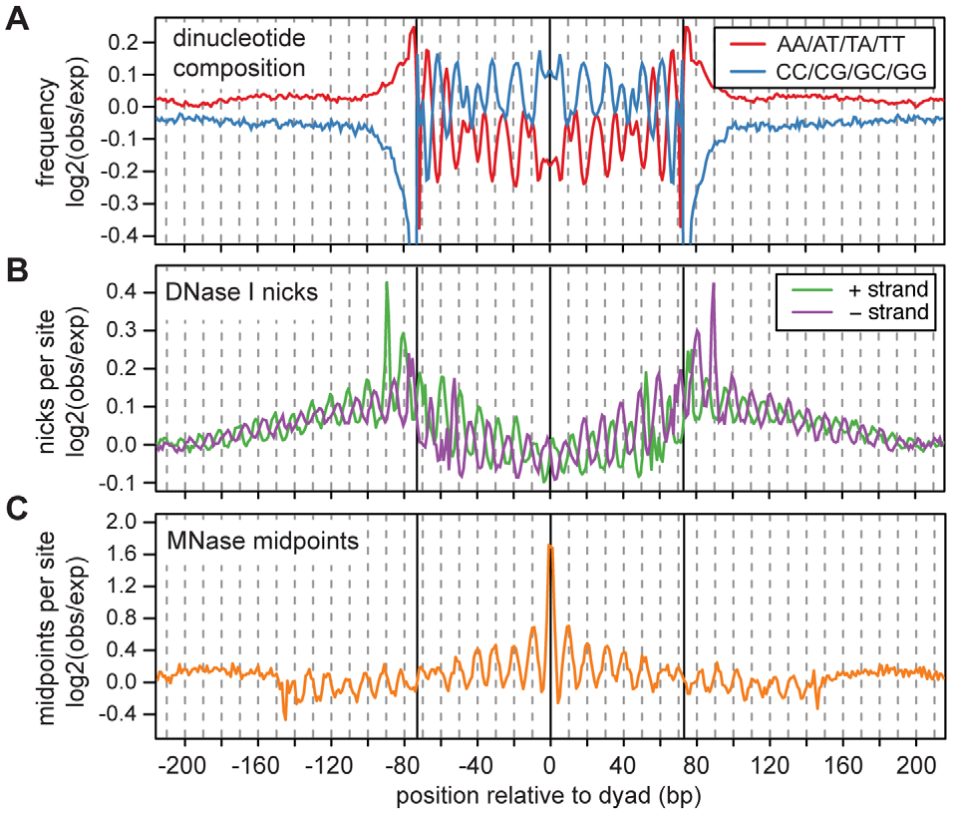
Fine scale characteristics of nucleosome sequences. A. Frequencies of AA/AT/TA/TT and CC/CG/GC/GG dinucleotides across nucleosome sequences normalized by expected dinucleotide frequencies (log2 ratio). Expected frequencies were taken from a set of simulated fragments, which were sampled such that they had the same MNase cutting bias as the observed fragments. B. DNase I cut rates across nucleosome sequences normalized by the expected cut rates (log2 ratio). Expected DNase I cut frequencies were estimated from the composition of all observed DNase I cut sites in the human genome. C. MNase-seq fragment midpoints from 3 cell lines. Expected midpoint frequencies were estimated from the same simulated fragments used in A.

We examined the nucleotide composition of the 147 bp fragments, and found clear 10 base pair periodicities in all 16 dinucleotides (Figure S2); this result is largely unaffected when we correct for MNase cutting bias (Figure 1A). The dinucleotide patterns closely resemble those found in other organisms [5], [15], [16], [18] and *in vitro* [7], [16], [17], and argue that these dinucleotides are important for the rotational positioning of human nucleosomes *in vivo*. Note that the aggregate dinucleotide pattern does not necessarily represent any single nucleosome sequence, but instead reflects a preference for dinucleotides to be placed at specific positions along the nucleosome. When we calculate dinucleotide frequencies using our single-end sequences, the periodic dinucleotide pattern is greatly attenuated (Figures S2, S3 and S4), which confirms that we attain greater precision through the use of paired-end reads.

We investigated the sensitivity of nucleosome sequences to nicking by the DNase I enzyme using 3.0 billion experimentally identified DNase I nick sites [30]. Across the mapped 147 bp MNase fragments, there is a 10 base pair periodicity in DNase I nicking and a phase-shift of 2–3 bp between the two strands (Figure 1B). This pattern reflects the three dimensional structure of DNA that is wrapped around the nucleosome core (the minor groove is only accessible to DNase I when it faces outward from the histone surface [34]–[37]), and indicates that a substantial fraction of nucleosomes must have consistent rotational positioning [34].

Unexpectedly, the 10 bp periodicity in DNase I sensitivity extends beyond the putative nucleosome core region into the adjacent linker sequences. This suggests that nucleosomes in different cells often have translational offsets that are multiples of 10 bp. This would maintain their rotational positioning and result in a longer periodic pattern of DNase I sensitivity in aggregate. To look for further evidence of 10 bp offsets in translational positioning, we examined how other 147 bp MNase fragment midpoints are distributed around observed fragment midpoints. To avoid artifacts introduced by the MNase-seq protocol (such as duplicate sequences introduced by amplification), we ascertained midpoints using four cell lines, and examined the distribution of midpoints in the surrounding region using three other cell lines. This procedure reveals a striking periodic pattern, in which nucleosomes are much more likely to be positioned at “in-phase” distances that are multiples of 10 bp from the ascertained dyad (Figure 1C).

### Translational positioning of human nucleosomes

**Figure 2:**
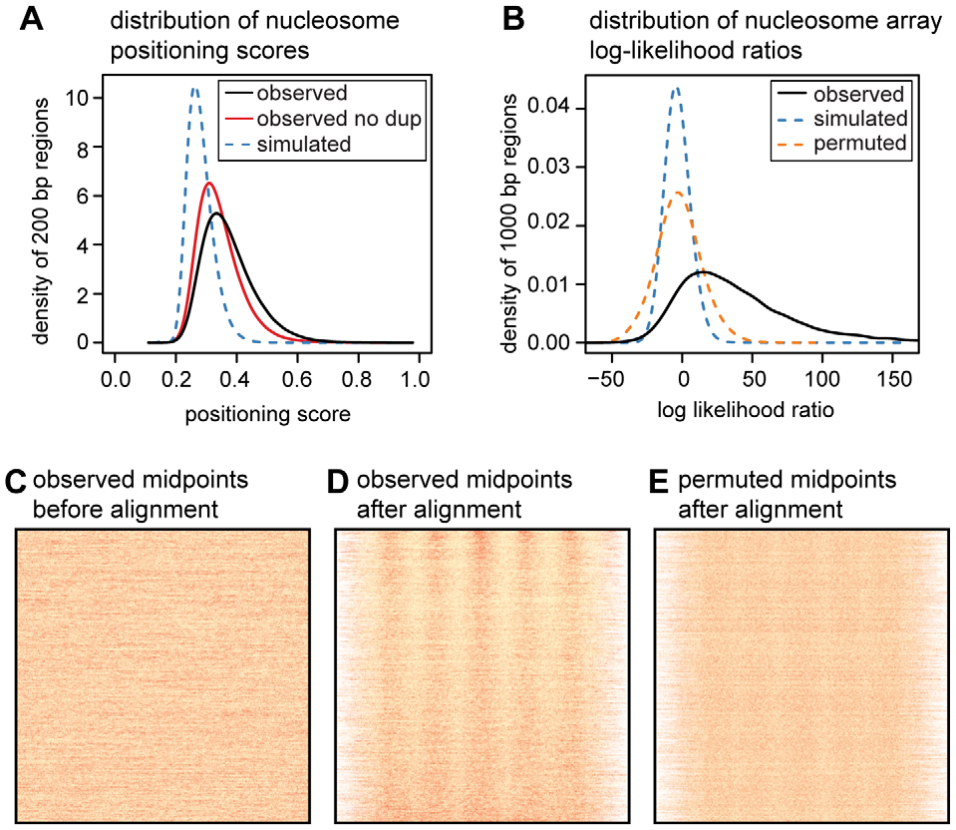
Quantifying translational nucleosome positioning in the human genome. A. Distribution of nucleosome positioning scores from a random sample of one million 200 bp regions (smoothed using a Gaussian kernel with bandwidth 0.01). Scores were also calculated in the same regions using midpoints from non-duplicate read pairs and from simulated read pairs. B. Distribution of nucleosome array log likelihood ratios (LLRs) for 23,763 randomly sampled 1 kb regions (smoothed using a Gaussian kernel with bandwidth 1.0). LLRs were also calculated using midpoints from simulated reads and using permuted versions of the same regions. C. Heatmap of MNase midpoints in the randomly sampled regions from B, prior to their alignment. D. Heatmap of MNase midpoints from panel D, after their alignment. Regions were aligned according to the most likely position of the central nucleosome. E. Heatmap of aligned MNase midpoints for permuted regions. Heatmaps in C, D, and E are ordered by the LLR of the observed midpoints.

To quantify the translational positioning of nucleosomes we calculated positioning scores for one million randomly sampled 200 bp regions. We define the positioning score for a particular site as the fraction of nearby midpoints (within 100 bp) that are within 15 bp of the site. The score for a given region is then the maximum score across all sites in the region. For the same regions we also calculated positioning scores using simulated midpoints (Figure 2A). The observed scores are skewed towards much higher values than those from simulated midpoints (mean of 0.37 vs. 0.28; p<2.2×10^−16^, two-sided Mann-Whitney U test), and we estimate that 84% of nucleosomes are more consistently positioned than expected by chance. We compute this estimate after conservatively filtering duplicate read pairs from each MNase-seq library (to avoid amplification artifacts) so the true fraction of nucleosomes with non-random positioning is likely to be even higher. For comparison, we also computed the nucleosome positioning stringency metric of Valouev *et al.* [14] using both our simulated and observed midpoints (Figures S5 and S6). By comparing the stringency values for real and simulated midpoints, we again estimate that 84% of nucleosomes have stronger positioning than expected by chance (note that in this comparison we did not filter duplicate reads).

While most nucleosomes have non-random positioning, the majority of translational positioning is weak. Using arbitrary score thresholds, we estimate that 81% of human nucleosomes have weak positioning (score between 0.3 and 0.5), 8.4% have moderately strong positioning (score between 0.5 and 0.7), and 0.3% have very strong positioning (score>0.7). This is a large enrichment over simulated midpoints where only 27%, 0.06% and 0.03% of regions meet these criteria (Table S2).

### Many nucleosomes are in regularly spaced arrays

We next sought to establish how much of the genome contains consistently positioned arrays of nucleosomes. We randomly sampled 100,000 genomic segments each 1 kb long and removed those where fewer than 80% of the sites were uniquely mappable. We split the remaining 47,528 regions into training and test data sets and estimated parameters for a probabilistic nucleosome array model from the training data. The array model specifies the probability of observing an MNase fragment midpoint at each position of an 879 bp “template” spanning 5 nucleosomes (Figure S7). Under this model, genomic regions with midpoints that match the phase and period of the template will yield the highest likelihoods. We then used a log likelihood ratio (LLR) to assess whether fragment midpoints in each of the 23,763 test regions were distributed uniformly or according to the template. By permuting midpoints in each region we are able to estimate a false discovery rate (FDR) for the log-likelihood ratio statistic. An LLR threshold of 27.8 corresponds to an FDR of 1%, and 53% of our sampled regions exceed this threshold.

As our LLR statistic compares a template model to a uniform model, some fraction of the significant regions may have non-uniform patterns that do not closely resemble nucleosome arrays. It is visually clear, however, that many of the high scoring regions contain evenly spaced arrays of nucleosomes, even those with an LLR close to the 1% FDR threshold (Figure S8). Even after removing the highest scoring nucleosome from each region, 47% of regions still contain a significant template match at an FDR of 1% (Figure S9). If we adopt a more conservative permutation procedure that retains two positioned nucleosomes in each region, we estimate the FDR for the same LLR threshold to be 19% (Figure S10). From these results we conclude that almost half of the genome contains nucleosome arrays, although the estimated proportion is sensitive to the method used and to the definition of what constitutes a nucleosome array.

To better understand what types of genomic regions contain consistently positioned nucleosome arrays we estimated the overlap of the significant arrays (LLR>27.8; FDR<1%) with chromatin states identified from histone marks in human LCLs [38]. Nucleosome arrays are enriched in active insulators, promoters and enhancers, but are depleted within actively elongating genes (Figure S11). However, the majority of regions with significant arrays (72%) are located in the heterochromatin state, which makes up most of the genome and is defined by an absence of histone modifications associated with gene activity, elongation, silencing or CTCF binding [38].

To investigate the contribution of DNA sequence to nucleosome organization within each of these chromatin states, we compared observed nucleosome occupancies to those predicted by an *in vitro* sequence model that incorporates steric exclusion of overlapping nucleosomes [33]. Observed and predicted nucleosome occupancies are correlated across the 47,527 sampled regions (Spearman's *ρ* = 0.69, p<2.2×10^−16^), but the strength of correlation varies widely (Figure S12). The correlation is lowest in the poised promoter (*ρ* = 0.12) and active promoter states (*ρ* = 0.37), and is highest in the weak transcription (*ρ* = 0.69) and heterochromatin (*ρ* = 0.72) states.

**Figure 3:**
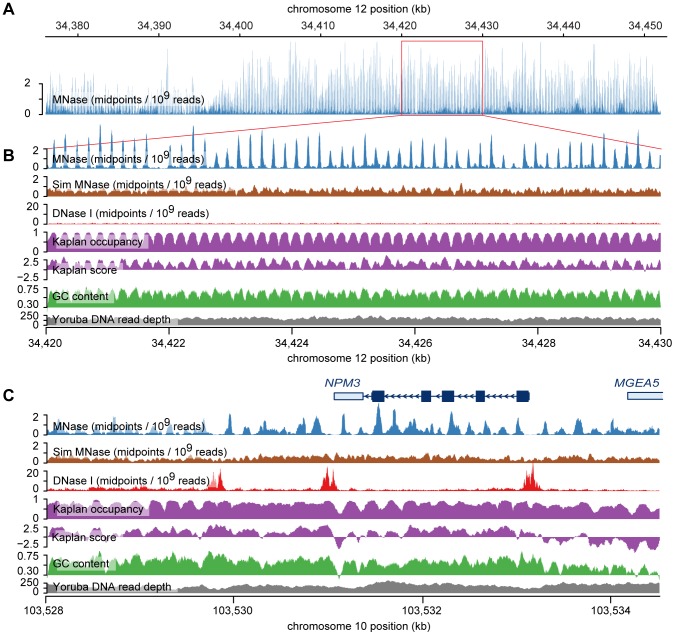
Examples of nucleosome arrays. A. MNase midpoint density (smoothed using a 30 bp sliding window) across a 76 kb region near the chromosome 12 centromere. This region contains an array of ∼400 nucleosomes with regular, consistent positioning. B. A small 10 kb subsection of the larger nucleosome array. Predicted nucleosome occupancy from the *in vitro* sequence model of Kaplan *et al.* [33] corresponds very well with MNase midpoint density. Kaplan scores predict the affinity of nucleosomes for the sequence but, unlike predicted occupancies, do not incorporate steric exclusion. DNase I nick density (smoothed with a 10 bp sliding window) indicates the location of DNase I sensitive regions (there are none in this region). The density of simulated MNase midpoints and Yoruba DNA sequencing read depth (aggregated across individuals from the 1000 genomes project) are not strongly correlated with MNase midpoint density, which shows that the array is not an artifact of sequencing or mapping bias. C. MNase midpoint density around the gene *NPM3*. In this region there is consistent, regular spacing of nucleosomes, but their positions are not well predicted by the Kaplan model, particularly in the DNase I hypersensitive sites, which are depleted of nucleosomes.

We also performed a scan for regions containing extremely strongly positioned arrays by sliding the nucleosome array template across the genome in 5 bp steps. Windows with low numbers of mapped fragments, or low mappability were removed and overlapping windows with likelihood ratios greater than 50 were merged. This genome-wide scan revealed many striking examples of regularly spaced, consistently positioned nucleosomes (Figure 3). Many of these arrays are adjacent to DNase I hypersensitive sites (Figure 3C), however strong nucleosome arrays also occur in regions without open chromatin. In these regions the DNA sequence itself appears to promote stable nucleosome configurations.

We observed a particularly extreme example of sequence-directed nucleosome positioning near the centromere of chromosome 12. This region spans ∼76 kb and contains over 400 consistently positioned nucleosomes in a single array (Figure 3A, 3B). This remarkable arrangement appears to result from a series of GC-rich sequences with high predicted nucleosome occupancy, which are separated by short AT-rich sequences with low predicted occupancy. This pattern resembles the ‘container elements’ described by Valouev *et al.* [14] and is created by a large number of degenerate tandem repeats that have periods of either ∼188 bp or ∼377 bp [39]. The periodicity in MNase midpoints does not appear to be an artifact of mappability in the region because the pattern is not observed in midpoints from simulated paired-end reads or in mapped reads from The 1000 Genomes Project (Figure 3B). In aggregate we observe elevated rates of DNase I nicking in the linker sequences of this region, as expected for strongly positioned nucleosomes (Figure S13). We estimate the nucleosome repeat length of the region to be 187 bp, which is slightly shorter than our genome-wide estimate for LCLs (192 bp) (Figure S14). This is also slightly shorter than the nucleosome repeat lengths of granulocytes (193 bp) and CD4+ T cells (203 bp), but matches or exceeds the repeat lengths of some chromatin domains in the latter cell type [14].

### Transcription factor binding sites are flanked by nucleosome arrays

We examined the positioning of nucleosomes around transcription factor binding sites, using publicly available chromatin immunoprecipitation followed by sequencing (ChIP-seq) data for 35 different transcription factors in LCLs [28]. So that we could identify nucleosome arrays, while allowing for nucleosome-depleted regions of variable width and uncertain location, we split our nucleosome array template into two mirror-image halves (Figure S7). We then used each half to search for nucleosome arrays upstream and downstream of each ChIP-seq peak. To avoid confounding with the known organization of nucleosomes around core promoters [11], [14], [18], [23]–[25], we examined only those ChIP-seq peaks that were at least 1 kb from a known transcription start site. Although our template is symmetric, recent work suggests that nucleosome organization at many transcription factor binding sites is asymmetric [40], [41].

**Figure 4:**
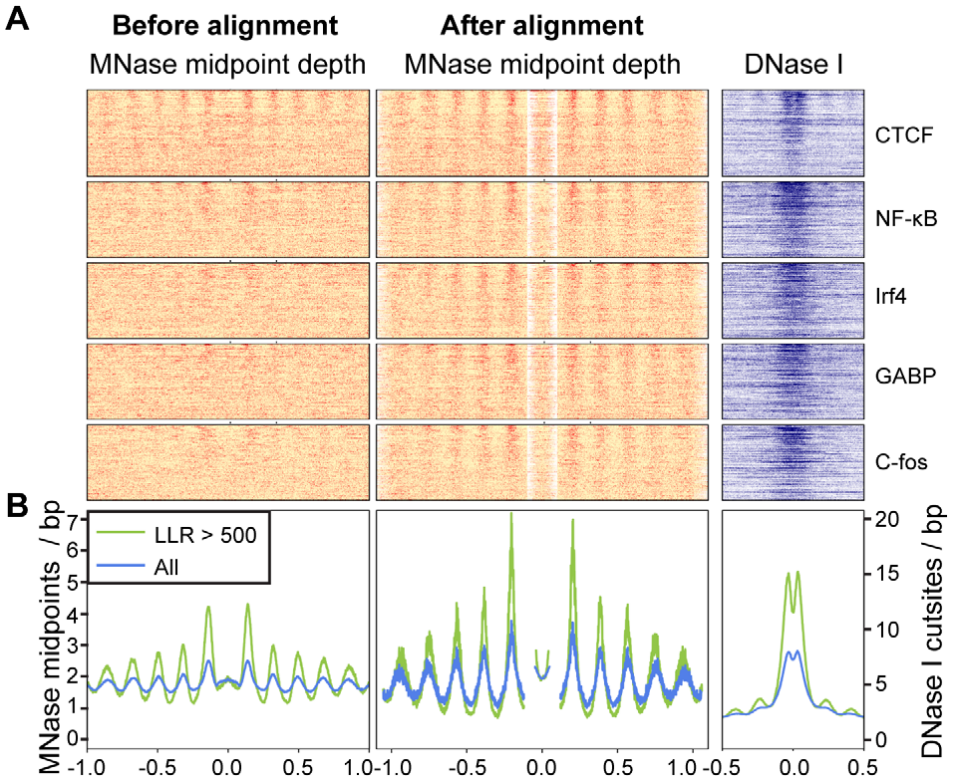
Arrays of positioned nucleosomes flanking transcription factor (TF) binding sites. A. Heatmaps of MNase midpoints (columns 1–2) and DNase I cuts (column 3) surrounding 1000 randomly sampled ChIP-seq peaks for CTCF, NF-kB, Irf4, GABP and C-fos. Heatmap rows are ordered from top to bottom by the nucleosome array log likelihood ratio (LLR). Columns 2 and 3 are aligned according to the most likely location of the upstream and downstream arrays of positioned nucleosomes. B. Aggregate MNase midpoint and DNase I cutsite depths across all regions and for the subset of regions with LLR>500.

**Figure 5:**
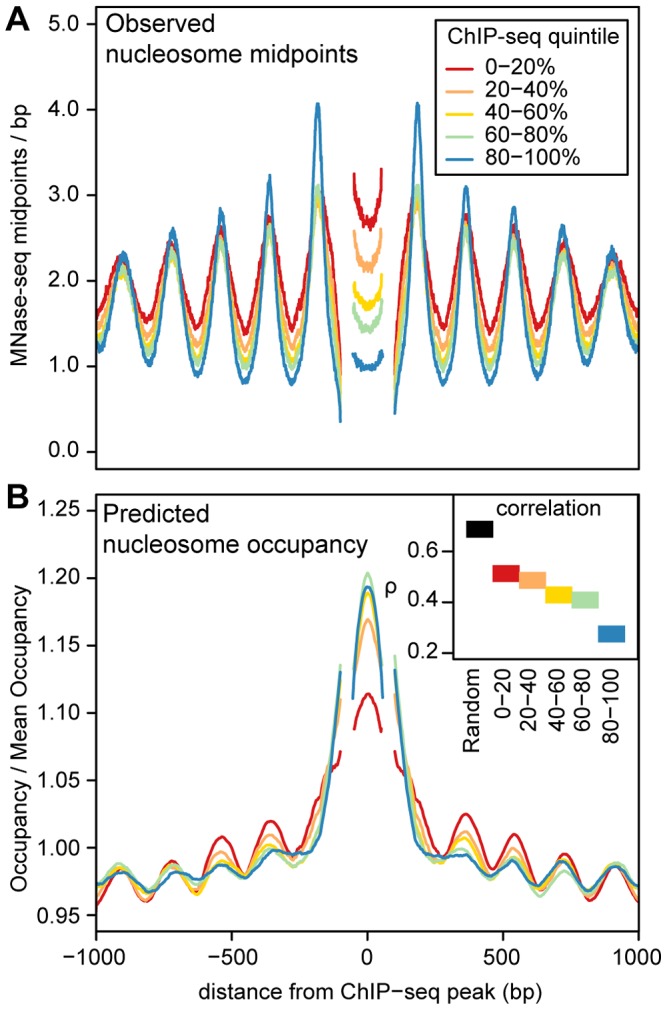
Predicted and observed nucleosome occupancy around ChIP–seq peaks. A. Mean MNase midpoint depth around ChIP-seq peak summits, aggregated across 5 transcription factors (CTCF, NF-kB, Irf4, C-fos and GABP). Regions are aligned such that the estimated locations of the +1 nucleosome, the −1 nucleosome and the midpoint between the nucleosomes are at the same position. Segments that have data from less than 50% of the ChIP-seq peaks (because of the variable spacing between nucleosomes) are omitted. Regions are stratified into ChIP-seq read depth quintiles, (higher quintiles indicate higher transcription factor occupancy). B. Predicted nucleosome occupancy from an *in vitro* sequence model [33]. Each region is normalized by the mean predicted occupancy of the entire region. As in A, regions are aligned on putative nucleosome positions and are stratified into ChIP-seq read depth quintiles and segments with data from less than 50% of the ChIP-seq peaks are omitted. The inset shows Spearman's rank correlation (*ρ*) between predicted and observed nucleosome occupancy for these regions and for 1000 randomly sampled genomic regions.

ChIP-seq peaks with strongly positioned flanking arrays are more sensitive to DNase I digestion and have far more pronounced DNase I footprints (Figure 4). As DNase I sensitivity is correlated with transcription factor occupancy [29], [30], we reasoned that transcription factors are more likely to influence nucleosome positions when their occupancy is high. To examine this relationship, we quantified transcription factor occupancy by ChIP-seq read depth. We ranked ChIP-seq peaks separately for each transcription factor and then aggregated data across transcription factors that fell into the same quintiles. For example, the top 20% of peaks for each factor were included in the same bin. As with DNase I sensitivity, transcription factor occupancy is closely related to the strength of nucleosome positioning (Figure 5A and Figure S15).

In the regions surrounding transcription factor binding sites, predicted occupancy is correlated with observed nucleosome occupancy (Figure 5B). This correlation diminishes with increasing transcription factor occupancy, which argues that the intrinsic sequence affinity of nucleosomes is overridden as transcription factor binding increases (Figure 5B). Even in the regions with the highest transcription factor occupancy, however, there is a detectable periodic pattern in aggregate predicted occupancy (Figure 5B). This pattern mirrors the observed nucleosome occupancy and suggests that nucleosome positions around bound transcription factors are not dictated solely by boundary effects, but are also partly encoded by the genome sequence. Another notable feature is that the ChIP-seq peaks are depleted of nucleosomes despite their high predicted occupancy (Figure 5). This is consistent with the previous observation that regulatory regions have high predicted nucleosome occupancy as a consequence of their high GC content [13], [42].

### Single nucleotide polymorphisms can affect the positioning of nucleosome arrays

The above results argue that nucleosome positions are guided by sequence preferences, which are frequently overridden by barriers that are sensitive to DNase I digestion. As a direct test of this hypothesis, we asked whether DNA sequence differences that affect DNase I sensitivity also affect nucleosome positions, using a set of 7088 DNase I sensitivity quantitative trait loci (dsQTLs) [30]. These are regions in which DNase I sensitivity is correlated with the genotype of a single nucleotide polymorphism (SNP). Frequently, the difference in DNase I sensitivity between genotypes is explained by SNPs that disrupt transcription factor binding sites [30].

**Figure 6:**
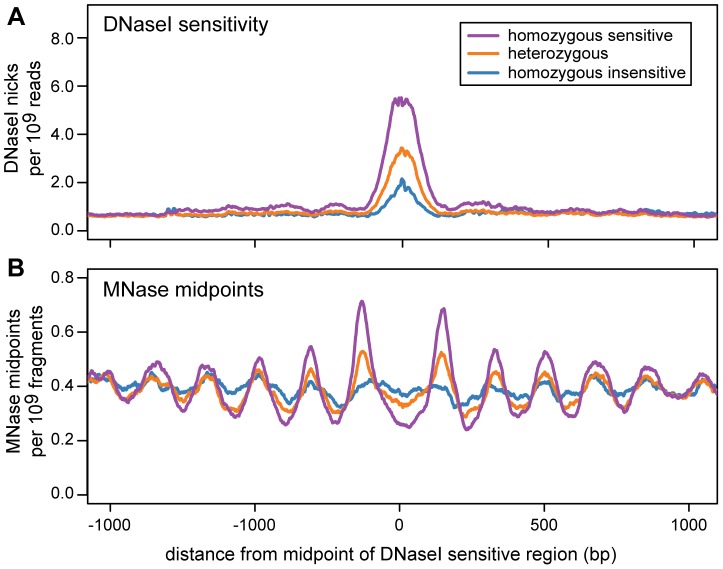
Nucleosome organization in regions with an association between DNase I sensitivity and genotype (dsQTLs). Data are aggregated across dsQTLs and are scaled by the total number of sequenced reads. The DNase-seq data are from 70 individuals and the MNase-seq data are from 7 individuals. This plot was created using a subset of dsQTLs (n = 1101) that have a narrow region of DNase I sensitivity (below the median) and a large difference in sensitivity between genotypes (above the median). The complete set of filtered dsQTLs shows the same trend (Figure S16). A. The density of DNase I nicks for different dsQTL genotypes. B. The density of MNase midpoints for different dsQTL genotypes.

For each dsQTL we classified each cell line as homozygous sensitive, heterozygous or homozygous insensitive, using the genotype of the associated SNP. We then examined the nucleosome organization of each genotype class by aggregating MNase-seq midpoints across dsQTLs. Regions that are homozygous for the sensitive genotype are flanked by arrays of positioned nucleosomes, consistent with those around ChIP-seq peaks (Figure 6; Figure S16). The strength of positioning is diminished in the heterozygous genotype, and is further reduced in the insensitive genotype (Figure 6).

## Discussion

Previous studies have found little evidence for consistent positioning of human nucleosomes [14]. Our data confirm that the translational positioning of most human nucleosomes is weak, but we also find that most nucleosomes are significantly more positioned than expected by chance. Additionally, a substantial fraction of nucleosomes have moderate or strong positioning.

At a fine scale, nucleosomes are often found at alternate “minor” translational positions that are multiples of 10 bp away from their most frequent “major” position. These alternate positions preserve the rotational positioning of the nucleosome on the DNA and are likely to be energetically favored because they retain phase with the periodic nucleosome sequence preferences. Similar offsets in nucleosome positions have been observed in 5S rDNA *in vitro* [43], [44] and are consistent with a weak 10 bp periodicity in MNase-seq reads from *C. elegans* [45]. Recently, this finding has been confirmed by chemical mapping of nucleosomes in yeast, which demonstrates that it is not an artifact of digestion by MNase [46].

At a broad scale, nucleosomes are often found in consistently positioned, regularly spaced arrays, which are enriched in insulators, promoters, and enhancers. These arrays frequently flank transcription factor binding sites and are strongest, in aggregate, when DNase I sensitivity and transcription factor occupancy are highest. Additionally, the consistent phasing of nucleosomes flanking DNase I sensitive sites is disrupted by single nucleotide polymorphisms that reduce DNase I sensitivity. This is strong evidence that the positioning of nucleosomes in these regions depends upon a barrier that is created by the binding of non-histone proteins to the DNA. A single nucleotide difference is unlikely to substantially change the affinity of a single nucleosome for a sequence, let alone shift the positions of multiple nucleosomes across a region spanning several thousand bases.

An interesting question is whether a barrier alone is sufficient to create arrays of regularly spaced nucleosomes. Recent results in yeast suggest that this may not be the case. While a minimal *in vitro* system can reconstitute a nucleosome free region and strongly-positioned +1 nucleosome near the transcription start site, flanking arrays are only formed in the presence of chromatin remodeling complexes and ATP [47], [48]. This argues that chromatin remodelers may pack nucleosomes against barriers or may increase the mobility of nucleosomes such that they can be pushed aside.

While the sequence preferences of nucleosomes are often overridden by other factors in functional regions of the genome, the abundance of nucleosomes with consistent rotational and translational positioning suggest that sequence preferences may play an important role in gene regulation. In particular, sequence-directed organization of nucleosomes may determine whether pioneer transcription factors that recruit chromatin remodelers can bind in the first place.

## Methods

### Cell lines and nuclei preparation

We studied *in vivo* nucleosome positioning in lymphoblastoid cell lines derived from seven Yoruba individuals obtained from Coriell (Table S1). Cell lines were cultured in RPMI 1640 media (supplemented with 2 mM L-glutamine and 15% fetal bovine serum) and maintained at a density of between 2–5×10^5^ viable cells/ml as per Coriell recommendations. Cells were pelleted at 1000 rpm at 4°C and washed in ice-cold PBS buffer. The cell pellet was resuspended in ice-cold NP-40 lysis buffer (10 mM Tris [pH 7.4], 10 mM NaCl, 3 mM MgCl_2_, 0.5% NP-40, 0.15 mM spermine, 0.5 mM spermidine) and incubated on ice for 5 minutes. The solution was centrifuged at 1000 rpm for 3 minutes at 4°C, and then washed in a wash buffer (10 mM Tris [pH 7.4], 15 mM NaCl, 60 mM KCl, 0.15 mM spermine, 0.5 mM spermidine), and centrifuged again at 1000 rpm for 3 minutes at 4°C. 1×10^8^ cells were then resuspended in 10 ml of ice-cold MNase digestion buffer (10 mM Tris [pH 7.4], 15 mM NaCl, 60 mM KCl, 0.15 mM spermine, 0.5 mM spermidine, 1 mM CaCl_2_).

### MNase digestion and DNA extraction

A 1 ml aliquot of nuclei was digested with 7 µl of 50 U/µl MNase at 37°C for 12 minutes. The reaction was stopped by addition of EDTA, SDS and NaCl to an end concentration of 0.01 M, 2% and 0.2 M respectively. Reactions were digested with RNaseA (0.1 mg) for 1 hr at 42°C and further treated with ProteinaseK at 37°C for one hour. DNA was extracted using phenol-chloroform extraction and concentrated by ethanol precipitation. DNA was then run on a 3.3% Nusieve agarose gel at 75 V for 5 hours. 147 bp fragments representing the mononucleosomes were excised from the gel and DNA was extracted from the gel by crushing the gel and soaking in soak buffer (300 mM Sodium Acetate, 1 mM EDTA, 0.1% SDS). The resulting DNA fragments in solution were then purified using a Qiagen PCR purification kit.

### Sequencing and read mapping

Our seven libraries of nucleosome fragments were prepared for paired-end sequencing using the standard Illumina protocol. Libraries were sequenced for a total of 36 or 50 cycles (18 bp or 25 bp for each end of the fragment) on either an Illumina Genome Analyzer II or Illumina HiSeq machine. For one flow cell only 25 bp single-end reads were generated due to a problem with the adaptor sequences. We retained the single-end reads for analysis, but also re-sequenced the two affected libraries to obtain a complete complement of paired-end data (Table S1).

We mapped reads to the hg18 assembly of the human genome using BWA (with default arguments) [49] and discarded alignments with mapping quality less than 10. We sorted and indexed alignments using samtools [50] and inferred the distribution of fragment sizes from the separation of read pairs. We discarded paired-end reads if their fragment size fell outside the central 95% of the fragment size distribution (126–184 bp). For paired-end reads the nucleosome dyad position was estimated to be the midpoint of the mapped fragment. For single-end reads, the dyad was assumed to be 75 bp downstream of the 5′ end of each mapped read because the median fragment size of the paired-end reads was 151 bp.

To estimate the number of mapped MNase-seq fragments per nucleosome, we assumed a genome size of 3 billion bases with one nucleosome every 200 bp.

A complete summary of the sequenced libraries is provided in Table S1.

### Fine-scale compositional properties of nucleosomes

To examine the fine scale properties of nucleosomes, we restricted our analysis to the 134 million mapped MNase-seq fragments of size 147 bp. To generate Figure 1, we calculated the mean dinucleotide composition, DNase I nick rate and density of MNase-seq fragment midpoints as a function of distance from the midpoints using a sample of 10% of these fragments. The locations of DNase I nicks across 70 lymphoblastoid cell lines were obtained from a previous study [30]. We calculated the density of MNase-seq fragment midpoints from 3 cell lines (GM19193, GM19238, GM19239) around midpoints ascertained using an independent set of 4 cell lines (GM18507, GM18508, GM18516, GM18522).

### Correction for mappability and MNase and DNase I cutting bias

MNase has a strong sequence specificity that could bias the positions of nucleosomes inferred using MNase-seq [51], [52] (although results obtained using a different enzyme suggest that this is not a substantial problem [53]). Mappability of reads could also affect our estimates of nucleosome positioning. To address both of these issues, we simulated reads and estimated the expected frequency of MNase midpoints at each position in the genome. Reads were simulated by randomly sampling genomic fragments with sizes drawn from the distribution of observed fragment sizes (Figure S1). We then performed rejection sampling of the fragments so that the 4-mer frequencies at their ends matched those of the real MNase-seq fragments (these positions have the strongest compositional bias (Figure S17)). Using this procedure we simulated 207 million pairs of 18 bp reads and 2.3 billion pairs of 25 bp reads. These numbers were chosen to approximately match the proportions of reads in the real data (Table S1). We mapped the simulated reads back to the genome using BWA and estimated the fragment sizes and midpoint locations using the same procedure that was employed for the real reads.

To correct for MNase cutting bias in Figure 1A, 1C and Figure S13 we calculated expected dinucleotide compositions and MNase-seq midpoint densities from the simulated 147 bp fragments. The simulated fragments were also used to estimate the expected distribution of nucleosome positioning scores and nucleosome array log-likelihood ratios as described below.

To correct for bias in DNase I nicking, we counted occurrences of 6-mers at observed DNase I nick sites. The 6-mers were extracted from positions −3 to +3 around each nick, which have the strongest compositional bias (Figure S18). We estimated an expected nicking rate for each 6-mer by dividing the number of times it occurs at a nick by the number times it occurs in the genome. We normalized observed DNase I nicking rates by dividing by the expected rate at each position (Figure 1B and Figure S13).

### Nucleosome positioning scores

To quantify the consistency of nucleosome positioning, we calculated positioning scores for a sample of one million 200 bp genomic regions. We only sampled from regions where at least 80% of the bases are uniquely mappable (defined by the wgEncodeDukeUniqueness24 bp track from the UCSC genome browser) and excluded regions that overlapped segments with excessive 1000 genomes read depth [54].

We defined the positioning score, *S*(*i*), at a genomic site *i* as the fraction of midpoints in a 201 bp window surrounding *i* that are within 15 bp of *i*:
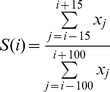
where *x_i_* is the number of midpoints at site *i*. For a given 200 bp region we define the score as the maximum score of all sites within the region. For this analysis, we used only MNase-seq fragments of size 142–152 bp and excluded regions that had fewer than 50 midpoints. In total 805,477 regions were retained for analysis. This score is similar to the stringency metric defined by Valouev *et al.* 2011 but we use a smaller window (201 bp rather than 301 bp) and do not perform kernel smoothing. For comparison, we also calculated stringency using the same method as Valouev *et al.* and obtained similar results (Figures S5 and S6). We also obtained very similar results when we calculated scores using 150 bp regions (instead of 200 bp) and using a window size of 151 bp (instead of 201 bp) (Figure S19).

As a control, we calculated positioning scores for midpoints from the simulated set of 142–152 bp fragments. To avoid amplification artifacts we also computed scores after conservatively removing duplicate read pairs from each MNase-seq library.

To calculate a FDR for positioning scores, we assigned each sampled region an empirical *p*-value (the fraction of scores from simulated midpoints that exceeded a region's score calculated from observed non-duplicate read pairs). We used the R package “q-value” [55] to calculate a false discovery rate corresponding to each possible *p*-value threshold and to estimate the proportion of nulls in the dataset, *π*_0_. We then estimated the fraction of nucleosomes showing more consistent positioning than expected by chance as 1-*π*_0_.

### Sliding window to detect arrays of positioned nucleosomes

We searched for well-ordered nucleosome arrays using a sliding window approach. In each window, we performed a likelihood ratio test that compared a model of a uniform distribution of MNase midpoints in the window (expected under no positioning) to an array model, where MNase midpoints are highly ordered into successive peaks and troughs. We modeled the spatial distribution of midpoints in the window as multinomial distribution, such that the likelihood of the midpoints in a window of *k* nucleotides is:
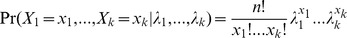
where *x*_i_ is the number of MNase midpoints observed at the *i*th nucleotide position of the window, *n* is the total number of midpoints, and *λ*_i_ is the probability that a midpoint is observed at position *i*. Under the null hypothesis of a uniform distribution of midpoints within the window *λ*_1_ = … = *λ*_k_ = *λ*_0_ = 1/*k*. For the alternative “nucleosome array” hypothesis, we used a set of empirically estimated midpoints probabilities (see below). Our likelihood ratio statistic was then:
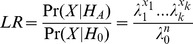
To scan for strongly positioned arrays of nucleosomes across the genome we used an 879 bp symmetric template spanning 5 nucleosomes. The template was initialized using the pattern of nucleosome positioning surrounding CTCF DNase I footprints from [30] and iteratively learned by aligning a training data set of 23,763 randomly selected 1 kb regions of high mappability. We computed log likelihood ratios (LLRs) for successive windows, advancing the template 5 bp each time and removing all windows with LLR<50, or with fewer than 879 fragment midpoints. Overlapping windows that remained after filtering were merged.

To estimate the fraction of the genome that contained nucleosome arrays, we selected 1 kb windows at random from the genome. For each window we slid a symmetric template over central 200 bp of the window in 1 bp increments computing a LLR for each successive step. Our procedure started with the template midpoint at a position 100 bp upstream of the window midpoint and ended with the template midpoint 200 bp downstream of the window midpoint. For each template we used all data in the 1 kb region to compute the LLR.

To align MNase data flanking ChIP-seq peaks we extracted MNase data from 2 kb windows flanking each ChIP-seq peak summit. To identify arrays of nucleosomes upstream and downstream of the peak summit we used two 1 kb nucleosome array templates separated by a nucleosome free region with a size of up to 200 bp. The probability of observing nucleosome midpoints in the nucleosome free region was assumed to be uniform with a rate equal to the mean number of midpoints per site in the region. For each window we estimated the size of the central nucleosome free region to be the one that gave the maximum likelihood. We then aligned all of the regions on the edges of their nucleosome free regions (Figure 4).

We investigated the performance of our array searching method in two ways. First, to assess the effect of mappability and MNase digestion biases we searched for nucleosome arrays in the simulated read data set described above. We extracted simulated reads for each of the 23,763 random 1 kb regions and performed the same array search procedure as for the real data. The distribution of LLRs from simulated read data is shown in Figure 2. Second, we estimated the proportion of “arrays” that may be driven by a single positioned nucleosome (Figure S9). For each of the 26,763 test regions we identified the most strongly positioned nucleosome in the array using a template containing a single positioned nucleosome. We removed this nucleosome and recomputed the LLR. This procedure was then repeated with a permuted data set from which we estimated the FDR.

### Chromosome 12 array analysis

To examine aggregate MNase midpoints and DNase I sensitivity in the chromosome 12 array region, we first identified locations of nucleosomes using the following procedure. We identified contiguous regions with nucleosome positioning scores>0.4 as ‘peak regions’ and labeled the position with the maximum score in each region as the peak. We discarded peaks where the score at the peak was less than 0.5, and when multiple positions tied we chose the one closest to the midpoint of the region. Using this method we identified 403 putative nucleosomes within the nucleosome array region (chr12:34,376,000–34,452,000), and used these to construct the aggregate plot shown in Figure S13. The locations of MNase midpoint peaks within the aggregate plot was used to estimate the nucleosome repeat length (Figure S14). We used the same procedure to estimate the genome-wide nucleosome repeat length, after aggregating data across a random sample of 200,000 nucleosome score peaks.

### Identification of ChIP–seq peaks

We downloaded publicly available ChIP-seq data for 40 transcription factors that were generated for the ENCODE consortium by the Bernstein, Myers and Snyder groups [28]. We removed all reads that had a quality score<10 and called peak locations using MACs [56]. Five transcription factors had a very small number of called peaks and were excluded from our analyses.

### Nucleosome organization around dsQTLs

We obtained a list of 7088 DNase I sensitivity quantitative trait loci (dsQTLs) from [30]. For each dsQTL, we labeled each cell line as homozygous sensitive, heterozygous or homozygous insensitive based on the genotype of the associated SNP. Genotypes for the cell lines were previously imputed from the 1000 Genomes Project data using BimBam [30], [31], [57], [58].

To more precisely identify the DNase I sensitive region within each dsQTL, we combined DNase I nick counts from homozygous sensitive and heterozygous cell lines and smoothed them with a 101 bp sliding window. We used the smoothed values to define a “peak” and “sensitive region”. We defined the “peak” as the site with the maximum value within 200 bp of the dsQTL's midpoint and defined the “sensitive region” as the block of contiguous sites around each peak where values exceeded 1/2 the peak value.

We then filtered the dsQTLs as follows. First, we discarded dsQTLs where the edge of the sensitive region was more than 100 bp from the dsQTL's midpoint (n = 396). Second, we filtered dsQTLs where the sensitive region overlapped one from another nearby dsQTL (n = 1463). Finally we discarded dsQTLs where the DNase I values in the peak region were inconsistent with the original, broader dsQTL region. We considered the DNase I values to be inconsistent with the dsQTL if the mean value for the heterozygote was greater than that of the homozygous sensitive genotype, or if the mean value for the homozygous insensitive genotype was greater than either that of the heterozygous or homozygous sensitive genotype. In total 911 dsQTLs were discarded by this latter criterion leaving a total of 4318 for analysis.

To examine the nucleosome organization in the remaining dsQTLs, each region was centered on the midpoint of the identified sensitive region and MNase midpoints were aggregated across regions separately for each of the three possible genotype classes.

### Data availability

All MNase-seq data are deposited in GEO under accession number GSE36979 and are available at http://eqtl.uchicago.edu.

## Supporting Information

Figure S1 Distribution of MNase-seq fragment sizes. Fragment sizes for each paired-end sequencing library were inferred from the separation of read pairs, which were mapped to the human genome using BWA.
(TIF)

Figure S2 Dinucleotide frequencies for all 16 dinucleotides computed using 147 bp fragments from paired-end MNase-seq. Unlike Figure 1 in the main text, these plots are not corrected for MNase digestion bias.
(TIF)

Figure S3 Dinucleotide frequencies for all 16 dinucleotides computed using 147 bp fragments from a single MNase-seq library for cell line GM19193. This cell line was under-digested compared to the other cell lines (Figure S1), but still provides much more precise nucleosome positioning information compared to the single-end libraries (compare to Figure S4).
(TIF)

Figure S4 Dinucleotide frequencies computed from single-end MNase-seq reads. While 10 bp periodic patterns are still visible in the dinucleotide composition, they are greatly attenuated compared to those obtained from paired-end reads as shown in Figures S2 and S3.
(TIF)

Figure S5 Scatterplot showing the relationship between positioning scores and the stringency metric of Valouev *et al.* 2011. Positioning scores and stringency were calculated for the same 805,477 randomly sampled regions. The scores are well-correlated (*R* ^2^ = 0.74; p<2.2×10^−16^), but our positioning scores tend to be higher (mean 0.37 vs. 0.23; p<2.2×10^−16^ by two-tailed t-test).
(TIF)

Figure S6 Distribution of stringency values for 200 bp windows. Stringency values were calculated for 805,477 randomly sampled regions using the method described by Valouev *et al.* 2011. For comparison, stringencies were calculated using midpoints from observed and simulated reads.
(TIF)

Figure S7 Template models for nucleosome arrays. The master template was initially derived from from CTCF binding sites and then re-trained on a set of random sequence regions. The re-trained template was used to derive the 879 bp template for genome-wide searching, and the templates used to discover nucleosome arrays flanking ChIP-seq binding sites.
(TIF)

Figure S8 Examples of nucleosome arrays for different log-likelihood ratio (LLR) ranges. LLRs were calculated for a set of 23,763 “test” regions, as described in the main text. Each row of panels shows four 2 kb regions that were randomly sampled from test regions, within a specified LLR range (e.g., the regions in the top row have LLRs between 0 and 25). The LLRs were computed using the central 1000 bp of each region. For each region the observed MNase-seq fragment midpoints (smoothed with a 50 bp sliding window) and the predicted nucleosome occupancy from the *in vitro* sequence model of Kaplan *et al.* 2009 are plotted.
(TIF)

Figure S9 Nucleosome array LLR distributions after removing the most highly-scoring nucleosome. A. The distribution of LLRs calculated for the set of 23,763 “test” regions and in permuted data. In each region we removed the most strongly positioned nucleosome prior to LLR computation in both the real and permuted data. B. Correlation between the full array LLRs and the LLRs computed after dropping the highest scoring nucleosome.
(TIF)

Figure S10 Distributions of nucleosome array LLRs after permuting midpoints from all but two of the positioned nucleosomes. For each of the 23,763 “test” regions we computed a LLR for the real data and for two permuted versions of the same region. For the “random” permutation, midpoint counts were randomly shuffled over the entire region. For the “2 nucleosomes” permutation, two nucleosomes were randomly selected and excluded from the permutation, while midpoint counts were randomly permuted between the remaining sites in the region.
(TIF)

Figure S11 Nucleosome array enrichment by chromatin state. Chromatin states for lymphoblastoid cell lines were obtained from Ernst *et al.* 2011. Enrichments were calculated using the set of 1 kb regions with significant nucleosome array LLRs (FDR<1%).
(TIF)

Figure S12 Correlation between observed and predicted nucleosome occupancy by chromatin state. Predicted nucleosome occupancy was obtained from Kaplan *et al.* 2009. Chromatin states for lymphoblastoid cell lines were obtained from Ernst *et al.* 2011. Observed nucleosome occupancy was calculated as the mean midpoint density in a 150 bp sliding window. Confidence intervals (95%, obtained by bootstrap) are shown as black line segments.
(TIF)

Figure S13 Aggregate MNase and DNase I as a function of distance from estimated nucleosome dyad positions in the chromosome 12 array region. Data are aggregated over 403 putative nucleosome dyads in the region spanning chr12:34,376,000–34,452,000 and smoothed with a 10 bp sliding window. Both MNase and DNase I rates were normalized by expected rates estimated from simulated midpoints and nucleotide composition.
(TIF)

Figure S14 Estimates of nucleosome repeat lengths. We estimated nucleosome repeat lengths using distances between MNase midpoint peaks in aggregate plots for the chromosome 12 array region (Figure S12) and genome-wide. The nucleosome repeat length is estimated as the slope of the least squares best fit line.
(TIF)

Figure S15 Distribution of nucleosome array log likelihood ratios (LLRs) around ChIP-seq peaks. LLRs around ChIP-seq peaks were calculated using two 1000 bp regions, flanking a central region of variable width, as described in Methods. Separate distributions are plotted for ChIP-seq peaks with different levels of transcription factor occupancy (as described in Figure 5 in the main text). Distributions are also presented for the same regions after permuting the MNase-seq fragment midpoint counts, and for a random sample of genomic regions.
(TIF)

Figure S16 Nucleosome organization in regions with an association between DNase I sensitivity and genotype (dsQTLs). This figure is as described in Figure 6 of the main text, except that data are aggregated across all 4318 filtered dsQTLs, rather than a subset. A. The density of DNase I nicks for different dsQTL genotypes. B. The density of MNase-seq fragment midpoints for different dsQTL genotypes.
(TIF)

Figure S17 MNase digestion bias. This figure shows the frequency of nucleotides around the ends of MNase-seq fragments that mapped to chromosome 1. K-mers from the first and last four bases of each fragment (+1 to +4) were used to correct for MNase digestion bias, as described in Methods.
(TIF)

Figure S18 DNaseI cutting bias. This figure shows the frequency of nucleotides around the genomic locations of 3.0 billion DNase I nick sites. K-mers spanning positions −3 to +3 were used to correct for bias in DNase I nicking, as described in Methods.
(TIF)

Figure S19 Distributions of nucleosome positioning scores calculated from 150 bp regions. Scores were computed from the same sample of one million regions used in Figure 2, but regions were decreased in size to 150 bp from 200 bp (retaining the same midpoint). The distributions are smoothed using a Gaussian kernel with bandwidth 0.01. For comparison we also calculated scores using 151 bp windows rather than 201 bp windows.
(TIF)

Table S1 Summary of MNase-seq data for this study. MNase-seq reads for 7 Yoruba lymphoblastoid cell lines were generated using either an Illumina Genome Analyzer IIx or Illumina HiSeq 2000 sequencer. Read lengths were either 18 bp or 25 bp, and both single-end and paired-end reads were generated. This table summarizes the number of MNase-seq fragments that were retained following mapping and filtering for each sequencing library. The raw sequencing reads have been deposited in GEO under accession number GSE36979.
(XLS)

Table S2 Percentage of nucleosomes with weak, moderate, or strong translational positioning. Positioning scores were calculated for 805,477 randomly sampled regions of 200 bp that had at least 50 midpoints. The percentages indicate the number of regions that meet the specified scoring criteria for observed midpoints, observed midpoints from non-duplicate read pairs, and simulated midpoints.
(XLS)

## References

[1] Kornberg RD (1974) Chromatin structure: a repeating unit of histones and DNA. Science 184: 868–871. 4825889 10.1126/science.184.4139.868

[2] Kornberg RD, Lorch Y (1999) Twenty-five years of the nucleosome, fundamental particle of the eukaryote chromosome. Cell 98: 285–294. 10458604 10.1016/s0092-8674(00)81958-3

[3] John S, Sabo PJ, Thurman RE, Sung MH, Biddie SC, et al (2011) Chromatin accessibility pre-determines glucocorticoid receptor binding patterns. Nat Genet 43: 264–8. 21258342 10.1038/ng.759 PMC6386452

[4] Kaplan T, Li XY, Sabo PJ, Thomas S, Stamatoyannopoulos JA, et al (2011) Quantitative Models of the Mechanisms That Control Genome-Wide Patterns of Transcription Factor Binding during Early Drosophila Development. PLoS Genet 7: e1001290 doi:10.1371/journal.pgen.1001290. 21304941 10.1371/journal.pgen.1001290 PMC3033374

[5] Albert I, Mavrich TN, Tomsho LP, Qi J, Zanton SJ, et al (2007) Translational and rotational settings of H2A.Z nucleosomes across the Saccharomyces cerevisiae genome. Nature 446: 572–576. 17392789 10.1038/nature05632

[6] Kaplan N, Hughes TR, Lieb JD, Widom J, Segal E (2010) Contribution of histone sequence preferences to nucleosome organization: proposed definitions and methodology. Genome Biol 11: 140. 21118582 10.1186/gb-2010-11-11-140 PMC3156944

[7] Lowary PT, Widom J (1998) New DNA sequence rules for high affinity binding to histone octamer and sequence-directed nucleosome positioning. J Mol Biol 276: 19–42. 9514715 10.1006/jmbi.1997.1494

[8] Thåström A, Lowary PT, Widlund HR, Cao H, Kubista M, et al (1999) Sequence motifs and free energies of selected natural and non-natural nucleosome positioning DNA sequences. J Mol Biol 288: 213–229. 10329138 10.1006/jmbi.1999.2686

[9] Iyer V, Struhl K (1995) Poly(dA:dT), a ubiquitous promoter element that stimulates transcription via its intrinsic DNA structure. EMBO J 14: 2570–2579. 7781610 10.1002/j.1460-2075.1995.tb07255.x PMC398371

[10] Struhl K (1985) Naturally occurring poly(dA-dT) sequences are upstream promoter elements for constitutive transcription in yeast. Proc Natl Acad Sci USA 82: 8419–8423. 3909145 10.1073/pnas.82.24.8419 PMC390927

[11] Yuan GC, Liu YJ, Dion MF, Slack MD, Wu LF, et al (2005) Genome-scale identification of nucleosome positions in S. cerevisiae. Science 309: 626–630. 15961632 10.1126/science.1112178

[12] Field Y, Kaplan N, Fondufe-Mittendorf Y, Moore IK, Sharon E, et al (2008) Distinct modes of regulation by chromatin encoded through nucleosome positioning signals. PLoS Comput Biol 4: e1000216 doi:10.1371/journal.pcbi.1000216. 18989395 10.1371/journal.pcbi.1000216 PMC2570626

[13] Tillo D, Hughes TR (2009) G+C content dominates intrinsic nucleosome occupancy. BMC Bioinformatics 10: 442. 20028554 10.1186/1471-2105-10-442 PMC2808325

[14] Valouev A, Johnson SM, Boyd SD, Smith CL, Fire AZ, et al (2011) Determinants of nucleosome organization in primary human cells. Nature 474: 516–520. 21602827 10.1038/nature10002 PMC3212987

[15] Satchwell SC, Drew HR, Travers AA (1986) Sequence periodicities in chicken nucleosome core DNA. J Mol Biol 191: 659–675. 3806678 10.1016/0022-2836(86)90452-3

[16] Segal E, Fondufe-Mittendorf Y, Chen L, Thåström A, Field Y, et al (2006) A genomic code for nucleosome positioning. Nature 442: 772–778. 16862119 10.1038/nature04979 PMC2623244

[17] Widlund HR, Cao H, Simonsson S, Magnusson E, Simonsson T, et al (1997) Identification and characterization of genomic nucleosome-positioning sequences. J Mol Biol 267: 807–817. 9135113 10.1006/jmbi.1997.0916

[18] Mavrich TN, Jiang C, Ioshikhes IP, Li X, Venters BJ, et al (2008) Nucleosome organization in the Drosophila genome. Nature 453: 358–362. 18408708 10.1038/nature06929 PMC2735122

[19] Mavrich TN, Ioshikhes IP, Venters BJ, Jiang C, Tomsho LP, et al (2008) A barrier nucleosome model for statistical positioning of nucleosomes throughout the yeast genome. Genome Res 18: 1073–1083. 18550805 10.1101/gr.078261.108 PMC2493396

[20] Wasson T, Hartemink AJ (2009) An ensemble model of competitive multi-factor binding of the genome. Genome Res 19: 2101–2112. 19720867 10.1101/gr.093450.109 PMC2775586

[21] Cairns BR (2009) The logic of chromatin architecture and remodelling at promoters. Nature 461: 193–198. 19741699 10.1038/nature08450

[22] Kornberg RD, Stryer L (1988) Statistical distributions of nucleosomes: nonrandom locations by a stochastic mechanism. Nucleic Acids Research 16: 6677–6690. 3399412 10.1093/nar/16.14.6677 PMC338322

[23] Schones DE, Cui K, Cuddapah S, Roh TY, Barski A, et al (2008) Dynamic regulation of nucleosome positioning in the human genome. Cell 132: 887–898. 18329373 10.1016/j.cell.2008.02.022 PMC10894452

[24] Zhang Y, Moqtaderi Z, Rattner BP, Euskirchen G, Snyder M, et al (2009) Intrinsic histone-DNA interactions are not the major determinant of nucleosome positions in vivo. Nat Struct Mol Biol 16: 847–852. 19620965 10.1038/nsmb.1636 PMC2823114

[25] Sasaki S, Mello CC, Shimada A, Nakatani Y, Hashimoto SI, et al (2009) Chromatin-Associated Periodicity in Genetic Variation Downstream of Transcriptional Start Sites. Science 323: 401–4. 19074313 10.1126/science.1163183 PMC2757552

[26] Fu Y, Sinha M, Peterson CL, Weng Z (2008) The insulator binding protein CTCF positions 20 nucleosomes around its binding sites across the human genome. PLoS Genet 4: e1000138 doi:10.1371/journal.pgen.1000138. 18654629 10.1371/journal.pgen.1000138 PMC2453330

[27] Kaplan N, Moore I, Fondufe-Mittendorf Y, Gossett AJ, Tillo D, et al (2010) Nucleosome sequence preferences influence in vivo nucleosome organization. Nat Struct Mol Biol 17: 918–20 author reply 920–2. 20683473 10.1038/nsmb0810-918 PMC2969171

[28] ENCODE Project Consortium (2011) Myers RM, Stamatoyannopoulos J, Snyder M, Dunham I, et al (2011) A user's guide to the encyclopedia of DNA elements (ENCODE). PLoS Biol 9: e1001046 doi:10.1371/journal.pbio.1001046. 21526222 10.1371/journal.pbio.1001046 PMC3079585

[29] Pique-Regi R, Degner JF, Pai AA, Gaffney DJ, Gilad Y, et al (2011) Accurate inference of transcription factor binding from DNA sequence and chromatin accessibility data. Genome Res 21: 447–55. 21106904 10.1101/gr.112623.110 PMC3044858

[30] Degner JF, Pai AA, Pique-Regi R, Veyrieras JB, Gaffney DJ, et al (2012) DNase I sensitivity QTLs are a major determinant of human expression variation. Nature 482: 390–394. 22307276 10.1038/nature10808 PMC3501342

[31] Genomes Project Consortium (2010) Durbin RM, Abecasis GR, Altshuler DL, Auton A, et al (2010) A map of human genome variation from population-scale sequencing. Nature 467: 1061–1073. 20981092 10.1038/nature09534 PMC3042601

[32] Noll M (1974) Subunit structure of chromatin. Nature 251: 249–251. 4422492 10.1038/251249a0

[33] Kaplan N, Moore IK, Fondufe-Mittendorf Y, Gossett AJ, Tillo D, et al (2009) The DNA-encoded nucleosome organization of a eukaryotic genome. Nature 458: 362–366. 19092803 10.1038/nature07667 PMC2658732

[34] Boyle AP, Davis S, Shulha HP, Meltzer P, Margulies EH, et al (2008) High-resolution mapping and characterization of open chromatin across the genome. Cell 132: 311–322. 18243105 10.1016/j.cell.2007.12.014 PMC2669738

[35] Cousins DJ, Islam SA, Sanderson MR, Proykova YG, Crane-Robinson C, et al (2004) Redefinition of the cleavage sites of DNase I on the nucleosome core particle. J Mol Biol 335: 1199–1211. 14729337 10.1016/j.jmb.2003.11.052

[36] Klug A, Lutter LC (1981) The helical periodicity of DNA on the nucleosome. Nucleic Acids Research 9: 4267–4283. 6272202 10.1093/nar/9.17.4267 PMC327434

[37] Lutter LC (1979) Precise location of DNase I cutting sites in the nucleosome core determined by high resolution gel electrophoresis. Nucleic Acids Research 6: 41–56. 424299 10.1093/nar/6.1.41 PMC327672

[38] Ernst J, Kheradpour P, Mikkelsen TS, Shoresh N, Ward LD, et al (2011) Mapping and analysis of chromatin state dynamics in nine human cell types. Nature 473: 43–49. 21441907 10.1038/nature09906 PMC3088773

[39] Benson G (1999) Tandem repeats finder: a program to analyze DNA sequences. Nucleic Acids Research 27: 573–580. 9862982 10.1093/nar/27.2.573 PMC148217

[40] Kundaje A, Kyriazopoulou-Panagiotopoulou S, Libbrecht M, Smith CL, Raha D, et al (2012) Ubiquitous heterogeneity and asymmetry of the chromatin environment at regulatory elements. Genome Res 22: 1735–1747. 22955985 10.1101/gr.136366.111 PMC3431490

[41] Lai WK, Buck MJ (2010) ArchAlign: coordinate-free chromatin alignment reveals novel architectures. Genome Biol 11: R126. 21182771 10.1186/gb-2010-11-12-r126 PMC3046486

[42] Tillo D, Kaplan N, Moore IK, Fondufe-Mittendorf Y, Gossett AJ, et al (2010) High nucleosome occupancy is encoded at human regulatory sequences. PLoS ONE 5: e9129 doi:10.1371/journal.pone.0009129. 20161746 10.1371/journal.pone.0009129 PMC2817738

[43] Pennings S, Meersseman G, Bradbury EM (1991) Mobility of positioned nucleosomes on 5 S rDNA. J Mol Biol 220: 101–110. 2067009 10.1016/0022-2836(91)90384-i

[44] Dong F, Hansen JC, Van Holde KE (1990) DNA and protein determinants of nucleosome positioning on sea urchin 5S rRNA gene sequences in vitro. Proc Natl Acad Sci USA 87: 5724–5728. 2377610 10.1073/pnas.87.15.5724 PMC54400

[45] Valouev A, Ichikawa J, Tonthat T, Stuart J, Ranade S, et al (2008) A high-resolution, nucleosome position map of C. elegans reveals a lack of universal sequence-dictated positioning. Genome Res 18: 1051–1063. 18477713 10.1101/gr.076463.108 PMC2493394

[46] Brogaard K, Liqun X, Wang JP, Widom J (2012) A base pair resolution map of nucleosome positions in yeast. Nature 486: 496–501. 22722846 10.1038/nature11142 PMC3786739

[47] Zhang Z, Wippo CJ, Wal M, Ward E, Korber P, et al (2011) A packing mechanism for nucleosome organization reconstituted across a eukaryotic genome. Science 332: 977–980. 21596991 10.1126/science.1200508 PMC4852979

[48] Gkikopoulos T, Schofield P, Singh V, Pinskaya M, Mellor J, et al (2011) A Role for Snf2-Related Nucleosome-Spacing Enzymes in Genome-Wide Nucleosome Organization. Science 333: 1758–1760. 21940898 10.1126/science.1206097 PMC3428865

[49] Li H, Durbin R (2009) Fast and accurate short read alignment with Burrows-Wheeler transform. Bioinformatics 25: 1754–1760. 19451168 10.1093/bioinformatics/btp324 PMC2705234

[50] Li H, Handsaker B, Wysoker A, Fennell T, Ruan J, et al (2009) The Sequence Alignment/Map format and SAMtools. Bioinformatics 25: 2078–2079. 19505943 10.1093/bioinformatics/btp352 PMC2723002

[51] Chung HR, Dunkel I, Heise F, Linke C, Krobitsch S, et al (2010) The effect of micrococcal nuclease digestion on nucleosome positioning data. PLoS ONE 5: e15754 doi:10.1371/journal.pone.0015754. 21206756 10.1371/journal.pone.0015754 PMC3012088

[52] Dingwall C, Lomonossoff GP, Laskey RA (1981) High sequence specificity of micrococcal nuclease. Nucleic Acids Research 9: 2659–2673. 6269057 10.1093/nar/9.12.2659 PMC326883

[53] Allan J, Fraser RM, Owen-Hughes T, Keszenman-Pereyra D (2012) Micrococcal nuclease does not substantially bias nucleosome mapping. J Mol Biol 417: 152–164. 22310051 10.1016/j.jmb.2012.01.043 PMC3314939

[54] Pickrell JK, Gaffney DJ, Gilad Y, Pritchard JK (2011) False positive peaks in ChIP-seq and other sequencing-based functional assays caused by unannotated high copy number regions. Bioinformatics 27: 2144–2146. 21690102 10.1093/bioinformatics/btr354 PMC3137225

[55] Storey JD, Tibshirani R (2003) Statistical significance for genomewide studies. Proc Natl Acad Sci USA 100: 9440–9445. 12883005 10.1073/pnas.1530509100 PMC170937

[56] Zhang Y, Liu T, Meyer CA, Eeckhoute J, Johnson DS, et al (2008) Model-based analysis of ChIP-Seq (MACS). Genome Biol 9: R137. 18798982 10.1186/gb-2008-9-9-r137 PMC2592715

[57] Guan Y, Stephens M (2008) Practical issues in imputation-based association mapping. PLoS Genet 4: e1000279 doi:10.1371/journal.pgen.1000279. 19057666 10.1371/journal.pgen.1000279 PMC2585794

[58] Scheet P, Stephens M (2006) A fast and flexible statistical model for large-scale population genotype data: applications to inferring missing genotypes and haplotypic phase. Am J Hum Genet 78: 629–644. 16532393 10.1086/502802 PMC1424677

