# Supplementary figures and images for: Controls of Nucleosome Positioning in the Human Genome
Source: PLoS Genet. 2012 Nov 15;8(11):e1003036. doi: 10.1371/journal.pgen.1003036 (PMC3499251; doi:10.1371/journal.pgen.1003036)

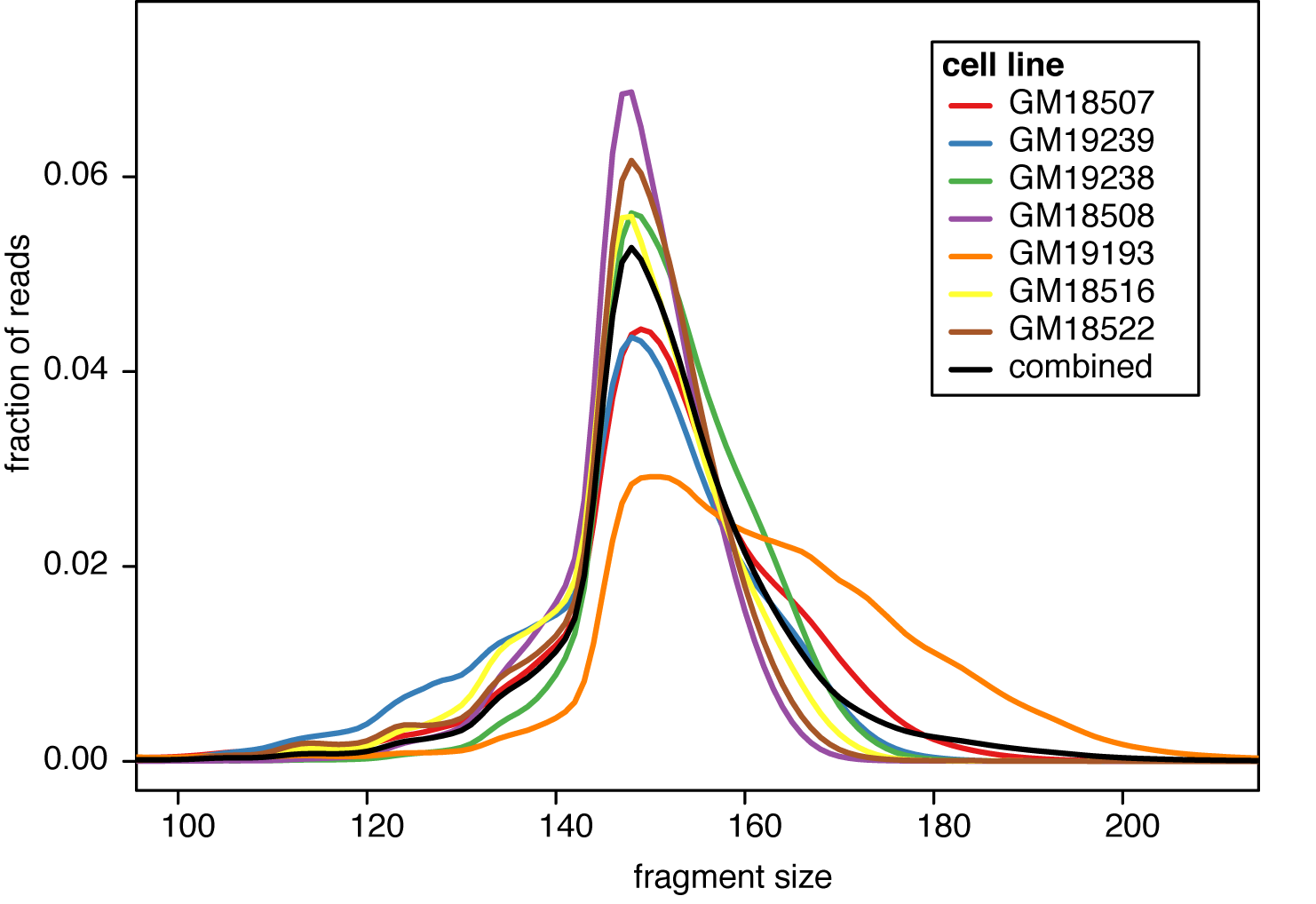

Supplement: Figure S1 — Distribution of MNase-seq fragment sizes. Fragment sizes for each paired-end sequencing library were inferred from the separation of read pairs, which were mapped to the human genome using BWA. (TIF) [file pgen.1003036.s001.tif]

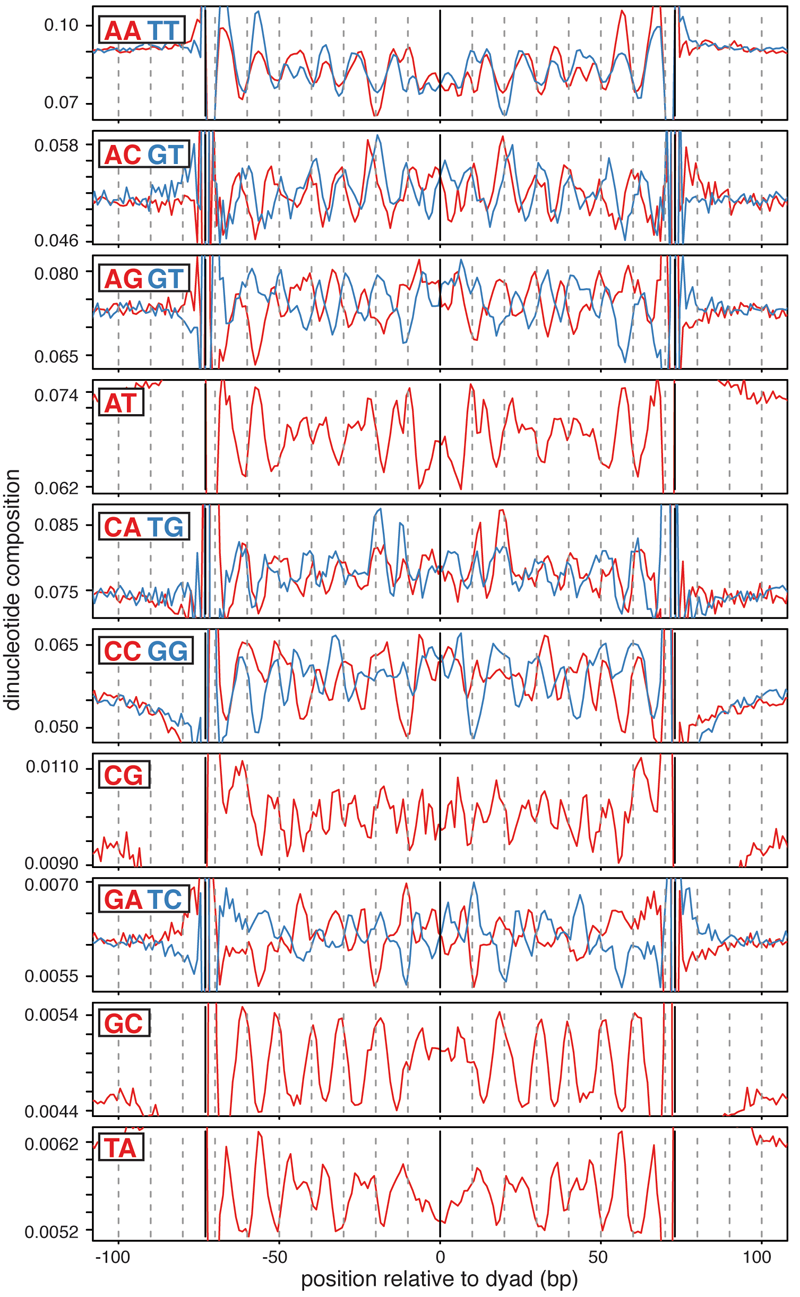

Supplement: Figure S2 — Dinucleotide frequencies for all 16 dinucleotides computed using 147 bp fragments from paired-end MNase-seq. Unlike Figure 1 in the main text, these plots are not corrected for MNase digestion bias. (TIF) [file pgen.1003036.s002.tif]

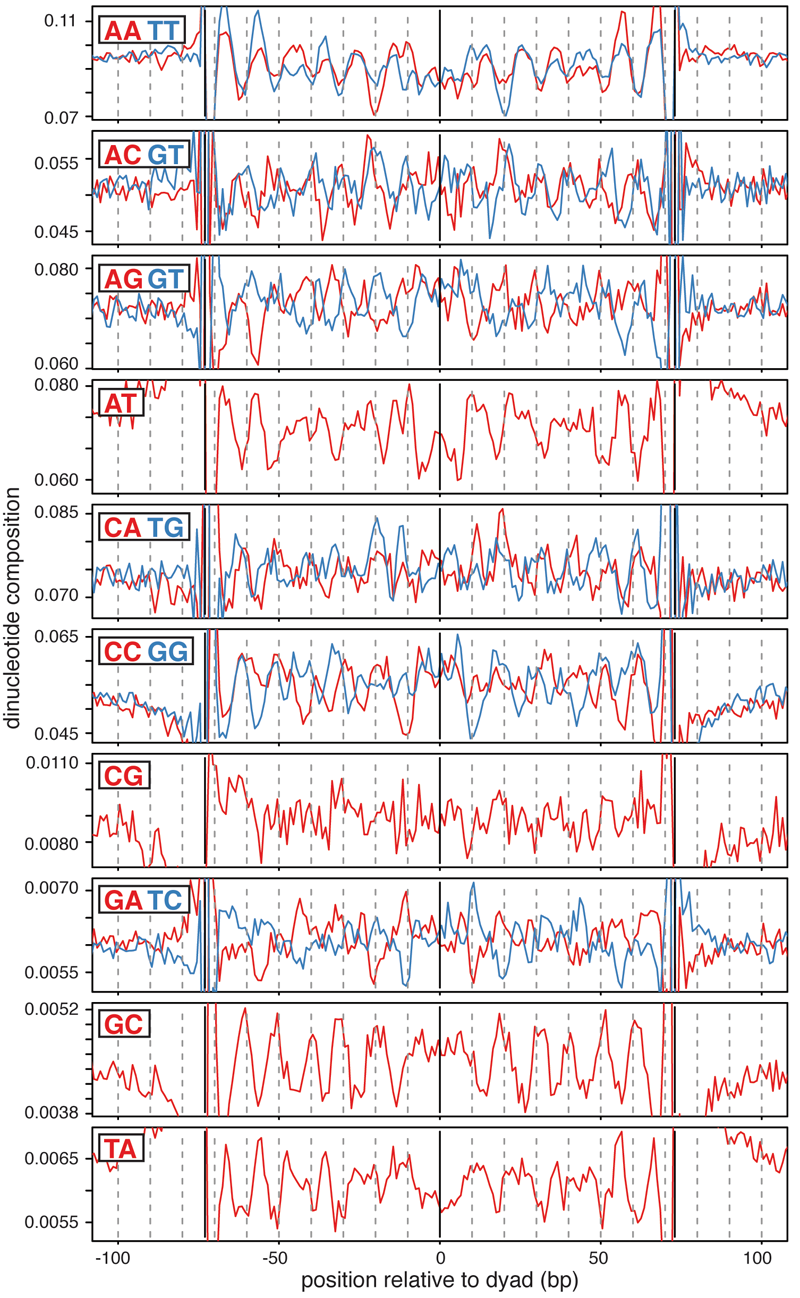

Supplement: Figure S3 — Dinucleotide frequencies for all 16 dinucleotides computed using 147 bp fragments from a single MNase-seq library for cell line GM19193. This cell line was under-digested compared to the other cell lines (Figure S1), but still provides much more precise nucleosome positioning information compared to the single-end libraries (compare to Figure S4). (TIF) [file pgen.1003036.s003.tif]

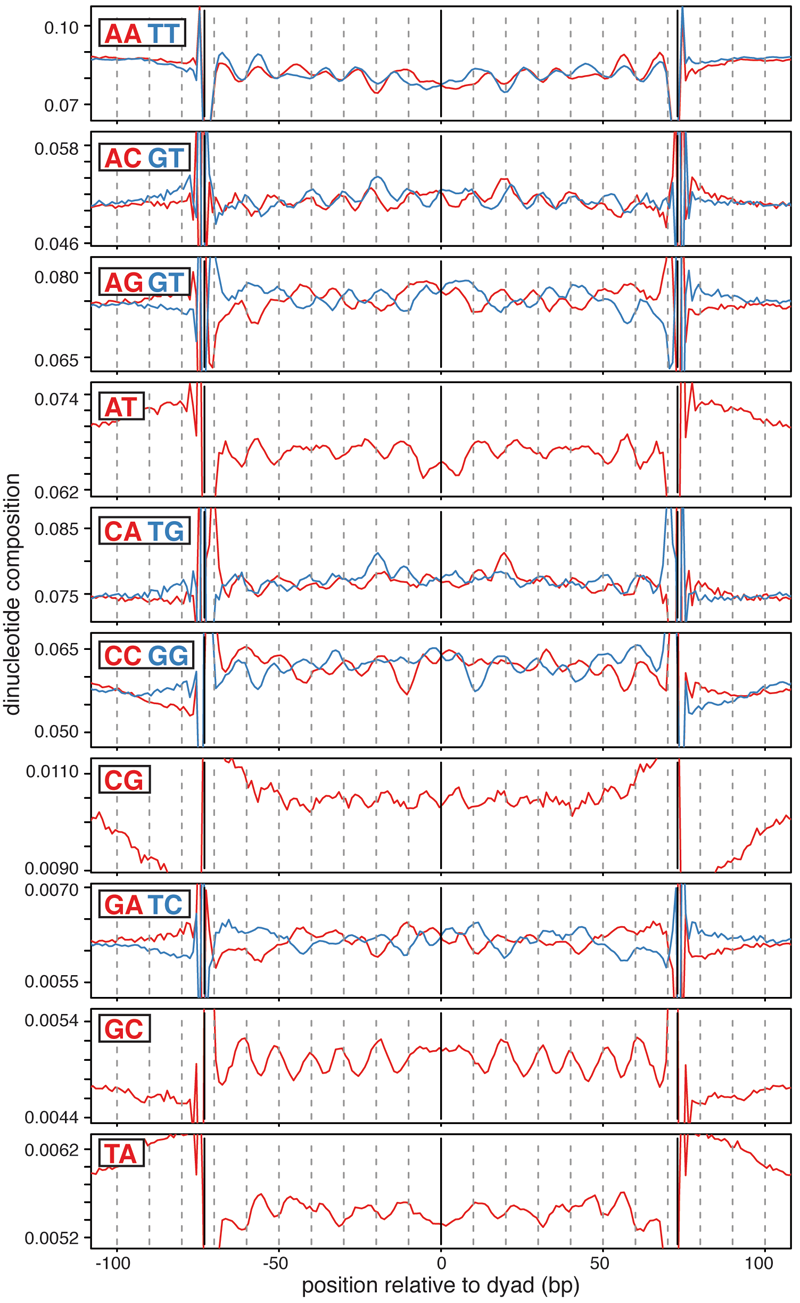

Supplement: Figure S4 — Dinucleotide frequencies computed from single-end MNase-seq reads. While 10 bp periodic patterns are still visible in the dinucleotide composition, they are greatly attenuated compared to those obtained from paired-end reads as shown in Figures S2 and S3. (TIF) [file pgen.1003036.s004.tif]

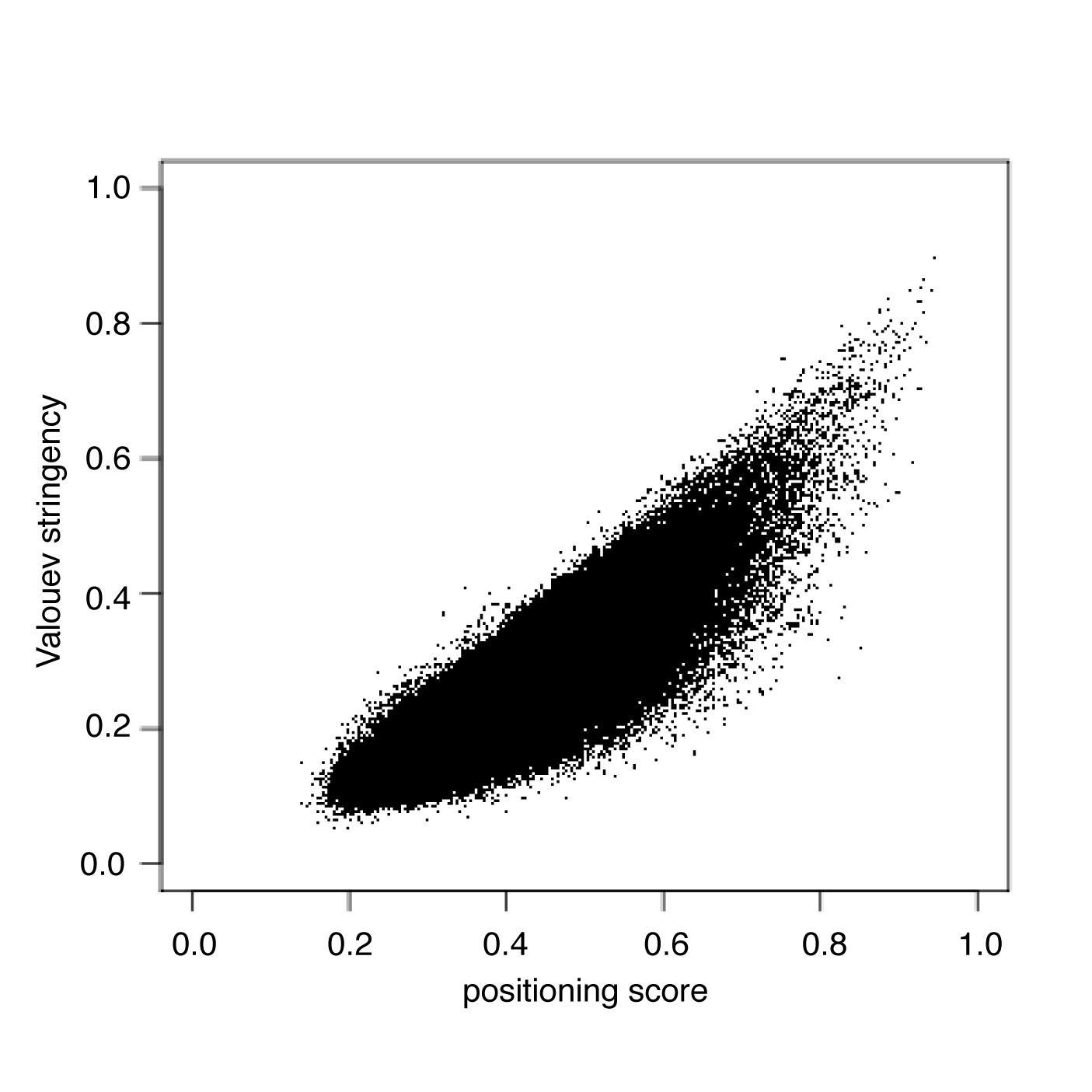

Supplement: Figure S5 — Scatterplot showing the relationship between positioning scores and the stringency metric of Valouev et al. 2011. Positioning scores and stringency were calculated for the same 805,477 randomly sampled regions. The scores are well-correlated (R 2 = 0.74; p<2.2×10^−16^), but our positioning scores tend to be higher (mean 0.37 vs. 0.23; p<2.2×10^−16^ by two-tailed t-test). (TIF) [file pgen.1003036.s005.tif]

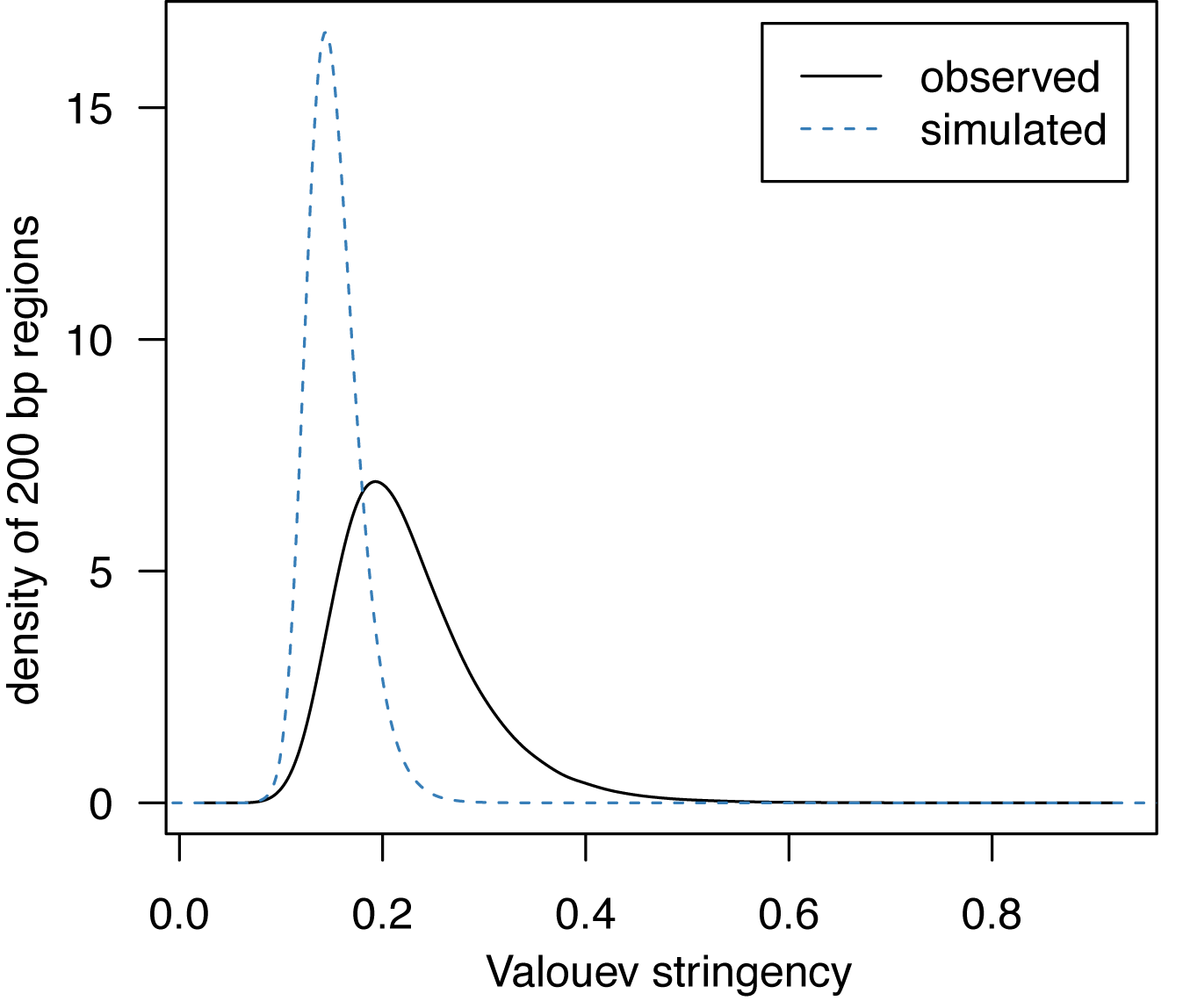

Supplement: Figure S6 — Distribution of stringency values for 200 bp windows. Stringency values were calculated for 805,477 randomly sampled regions using the method described by Valouev et al. 2011. For comparison, stringencies were calculated using midpoints from observed and simulated reads. (TIF) [file pgen.1003036.s006.tif]

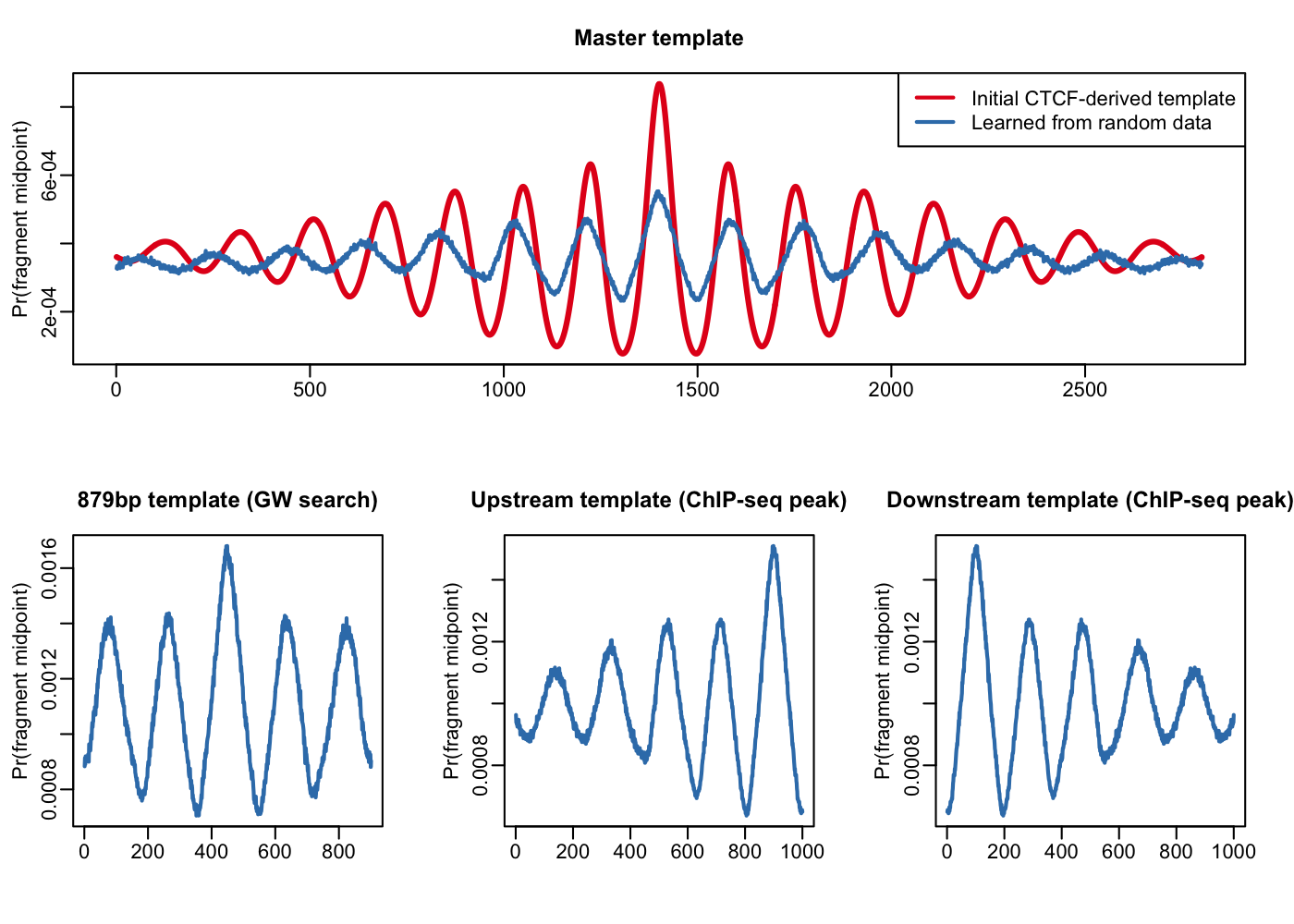

Supplement: Figure S7 — Template models for nucleosome arrays. The master template was initially derived from from CTCF binding sites and then re-trained on a set of random sequence regions. The re-trained template was used to derive the 879 bp template for genome-wide searching, and the templates used to discover nucleosome arrays flanking ChIP-seq binding sites. (TIF) [file pgen.1003036.s007.tif]

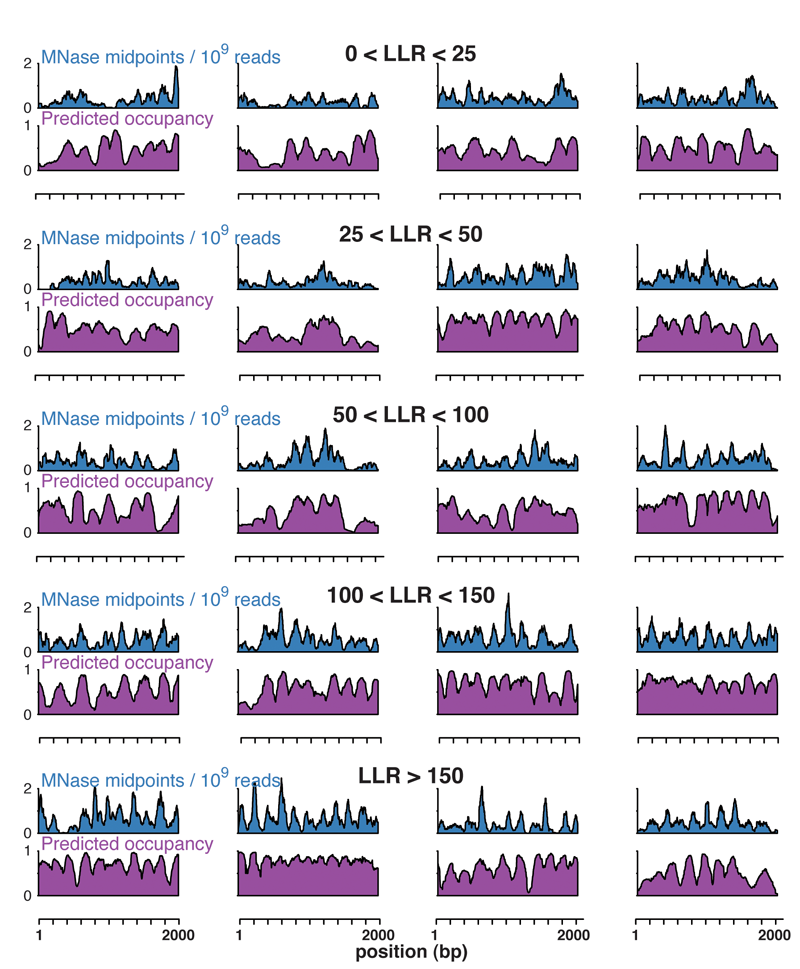

Supplement: Figure S8 — Examples of nucleosome arrays for different log-likelihood ratio (LLR) ranges. LLRs were calculated for a set of 23,763 “test” regions, as described in the main text. Each row of panels shows four 2 kb regions that were randomly sampled from test regions, within a specified LLR range (e.g., the regions in the top row have LLRs between 0 and 25). The LLRs were computed using the central 1000 bp of each region. For each region the observed MNase-seq fragment midpoints (smoothed with a 50 bp sliding window) and the predicted nucleosome occupancy from the in vitro sequence model of Kaplan et al. 2009 are plotted. (TIF) [file pgen.1003036.s008.tif]

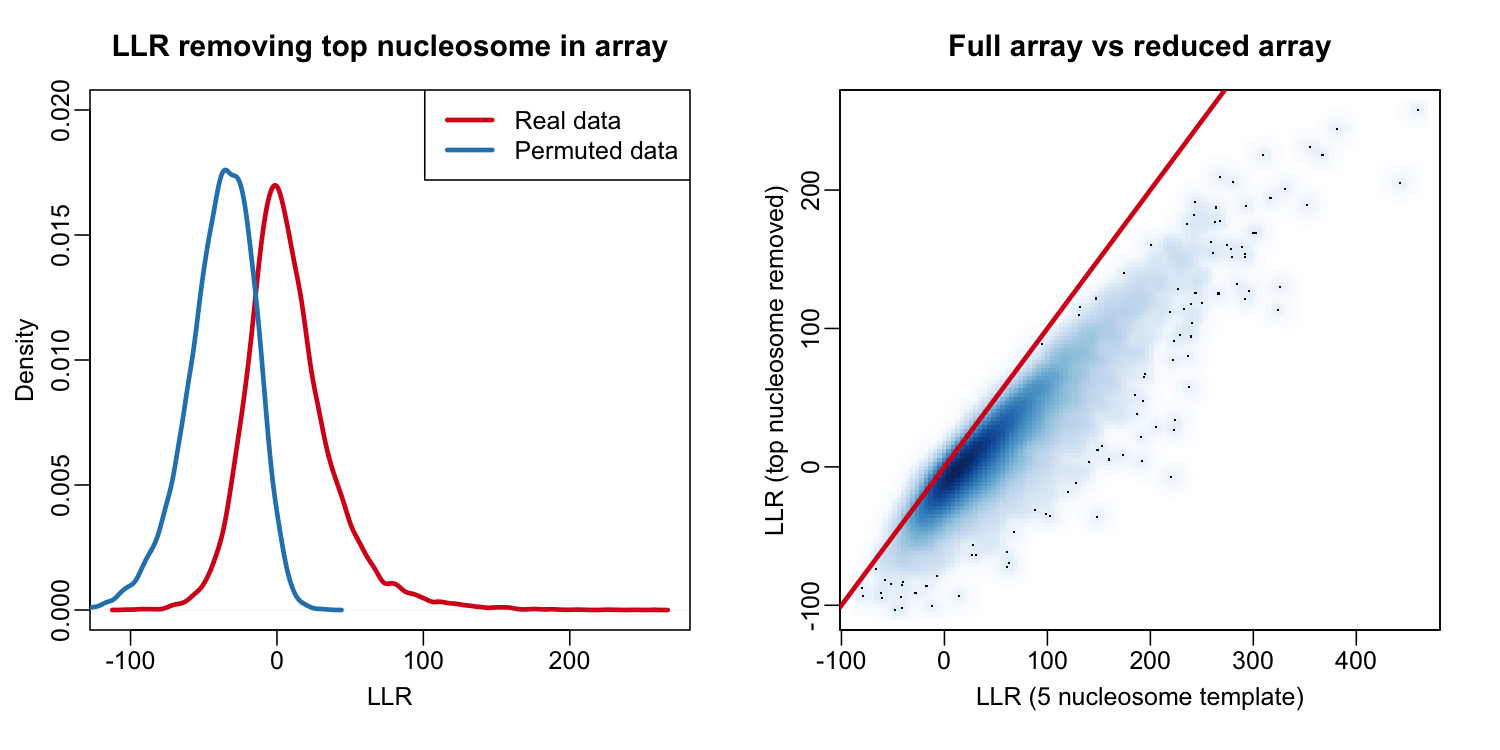

Supplement: Figure S9 — Nucleosome array LLR distributions after removing the most highly-scoring nucleosome. A. The distribution of LLRs calculated for the set of 23,763 “test” regions and in permuted data. In each region we removed the most strongly positioned nucleosome prior to LLR computation in both the real and permuted data. B. Correlation between the full array LLRs and the LLRs computed after dropping the highest scoring nucleosome. (TIF) [file pgen.1003036.s009.tif]

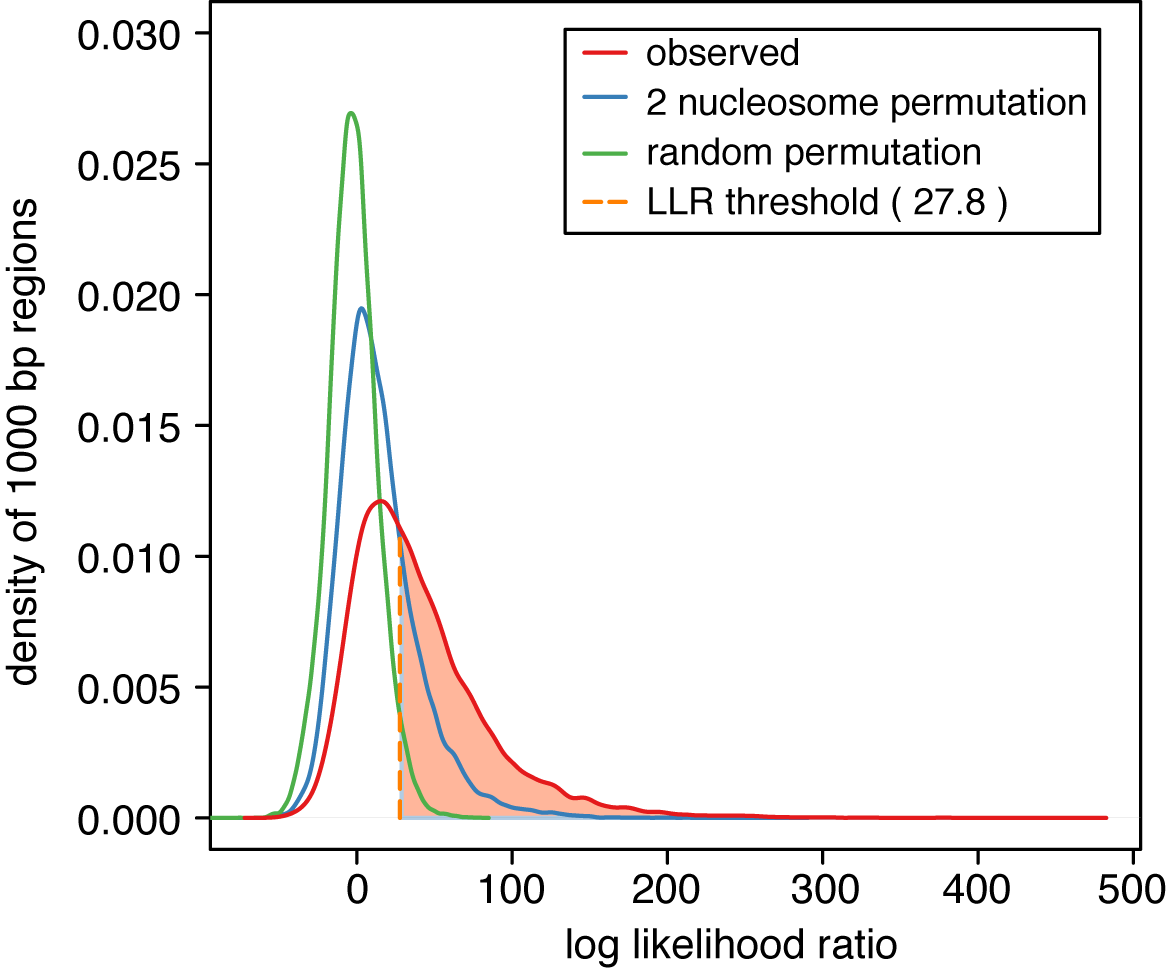

Supplement: Figure S10 — Distributions of nucleosome array LLRs after permuting midpoints from all but two of the positioned nucleosomes. For each of the 23,763 “test” regions we computed a LLR for the real data and for two permuted versions of the same region. For the “random” permutation, midpoint counts were randomly shuffled over the entire region. For the “2 nucleosomes” permutation, two nucleosomes were randomly selected and excluded from the permutation, while midpoint counts were randomly permuted between the remaining sites in the region. (TIF) [file pgen.1003036.s010.tif]

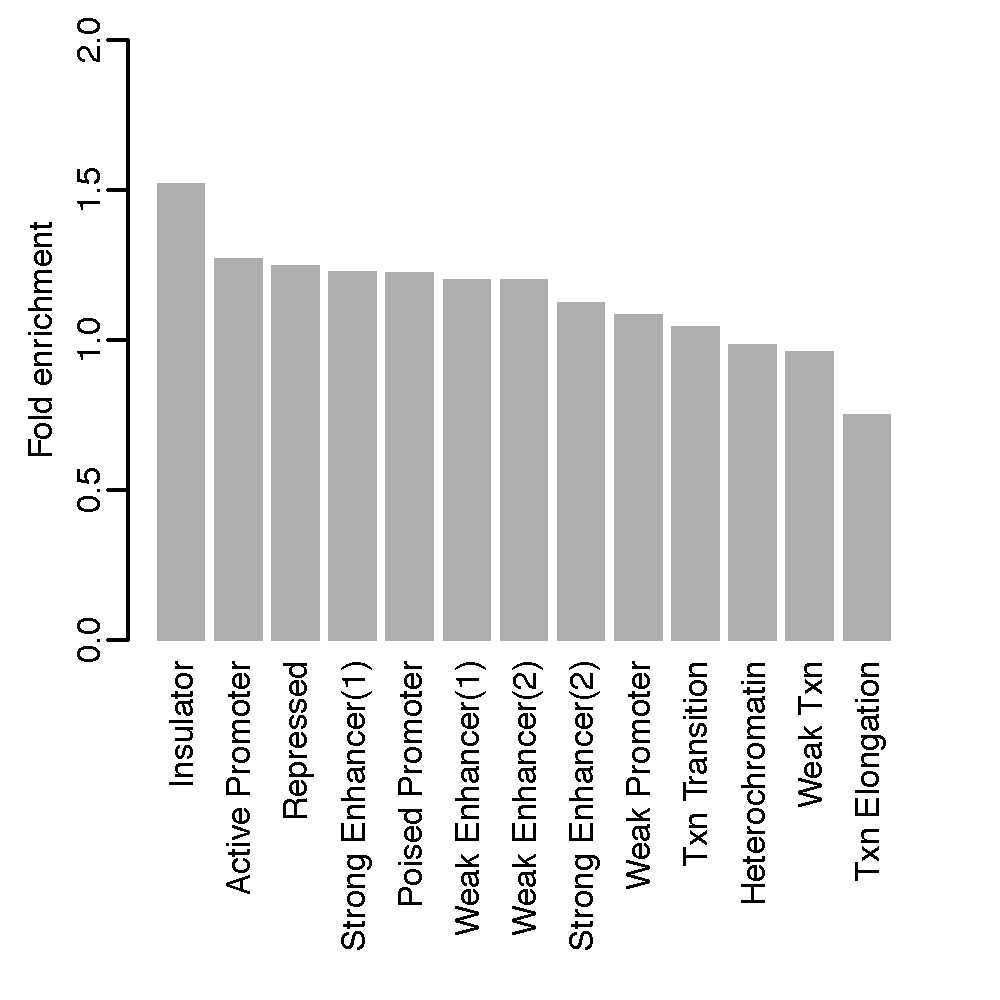

Supplement: Figure S11 — Nucleosome array enrichment by chromatin state. Chromatin states for lymphoblastoid cell lines were obtained from Ernst et al. 2011. Enrichments were calculated using the set of 1 kb regions with significant nucleosome array LLRs (FDR<1%). (TIF) [file pgen.1003036.s011.tif]

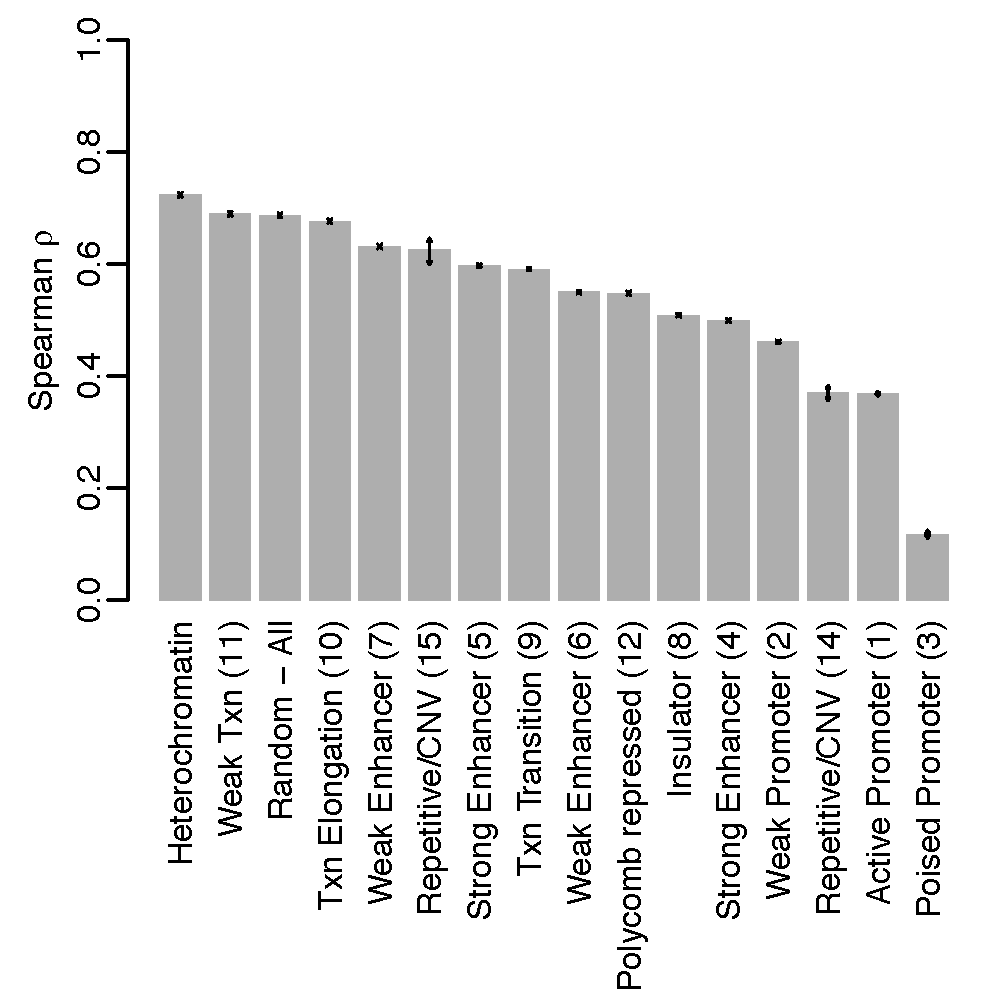

Supplement: Figure S12 — Correlation between observed and predicted nucleosome occupancy by chromatin state. Predicted nucleosome occupancy was obtained from Kaplan et al. 2009. Chromatin states for lymphoblastoid cell lines were obtained from Ernst et al. 2011. Observed nucleosome occupancy was calculated as the mean midpoint density in a 150 bp sliding window. Confidence intervals (95%, obtained by bootstrap) are shown as black line segments. (TIF) [file pgen.1003036.s012.tif]

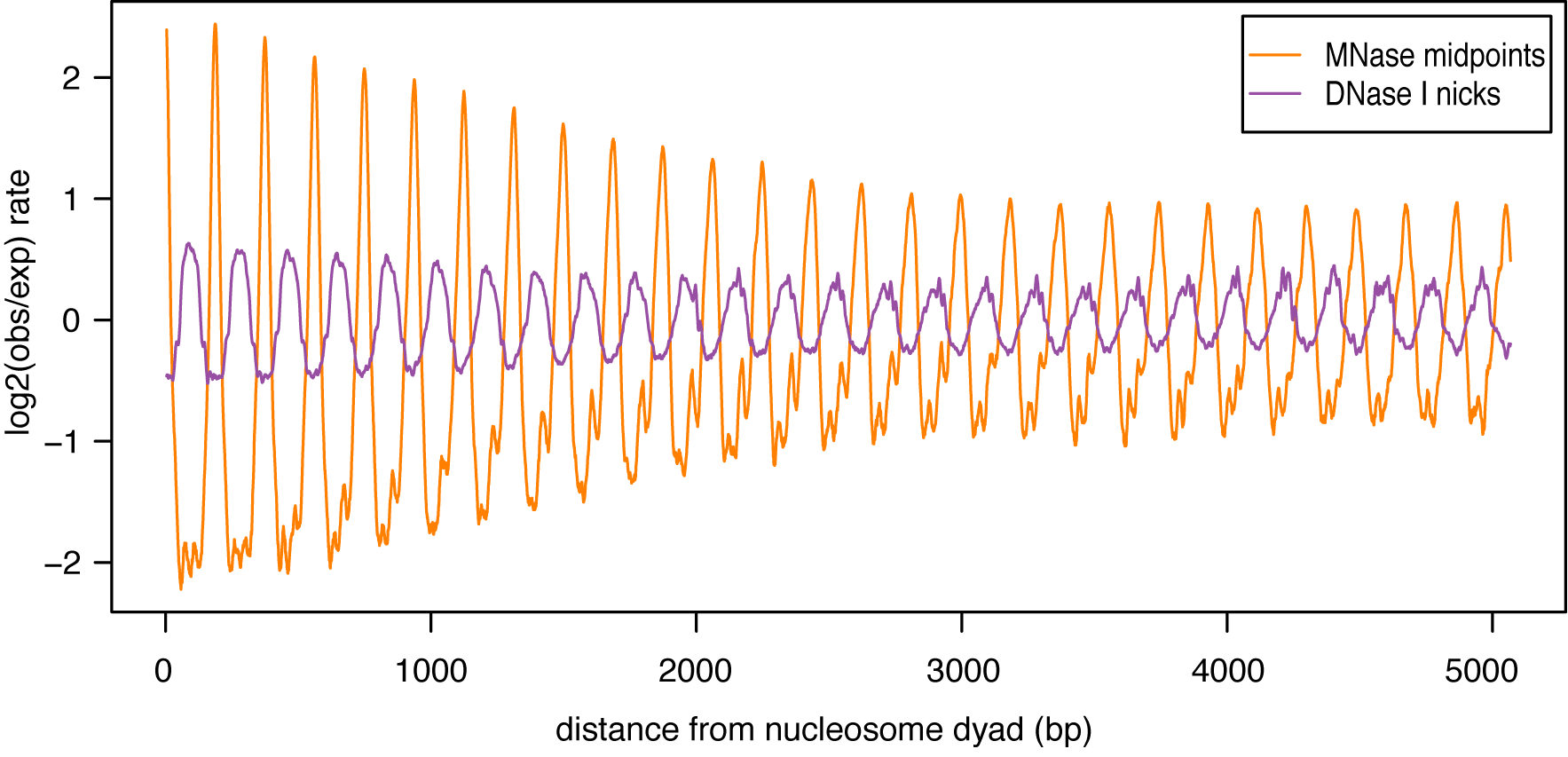

Supplement: Figure S13 — Aggregate MNase and DNase I as a function of distance from estimated nucleosome dyad positions in the chromosome 12 array region. Data are aggregated over 403 putative nucleosome dyads in the region spanning chr12:34,376,000–34,452,000 and smoothed with a 10 bp sliding window. Both MNase and DNase I rates were normalized by expected rates estimated from simulated midpoints and nucleotide composition. (TIF) [file pgen.1003036.s013.tif]

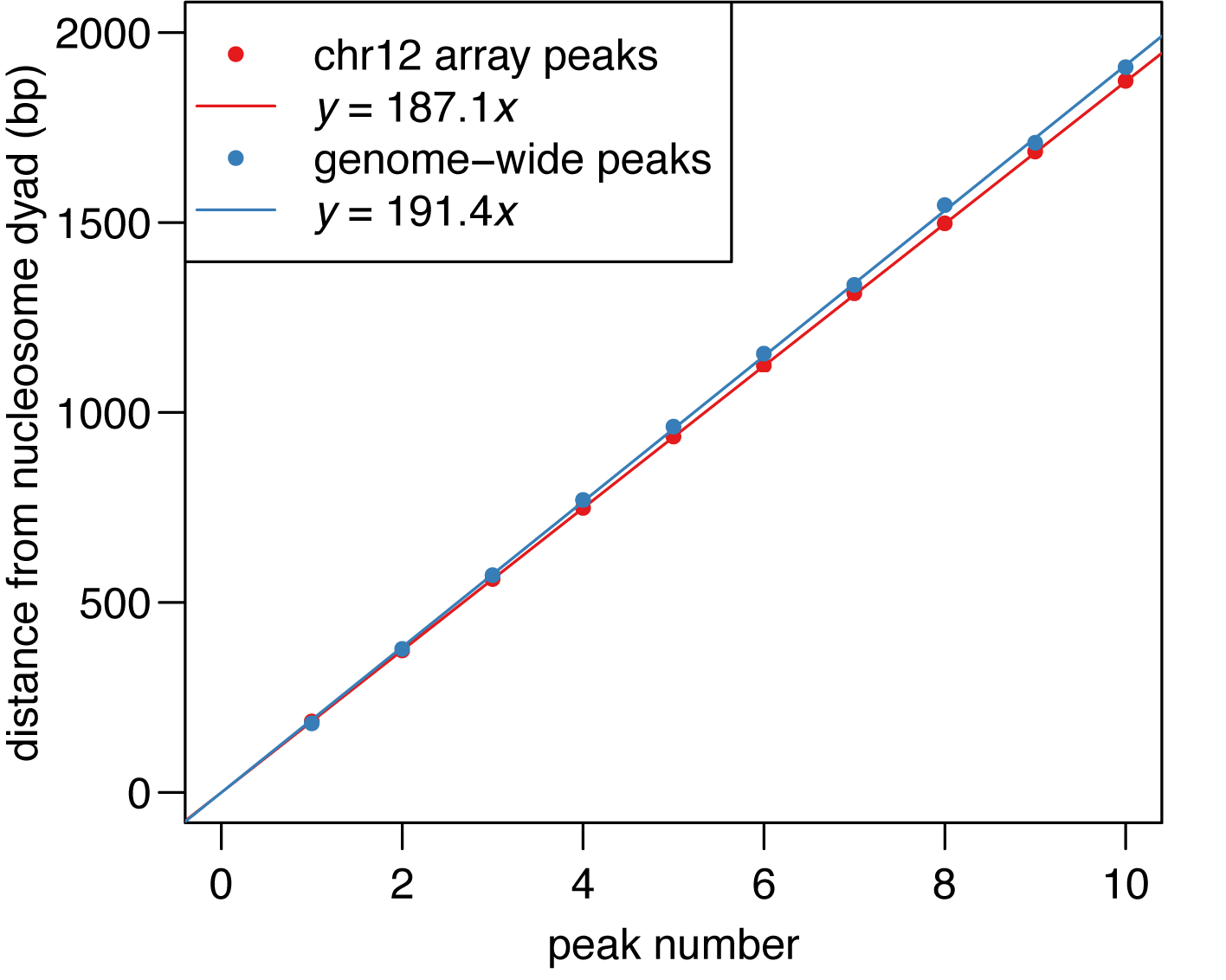

Supplement: Figure S14 — Estimates of nucleosome repeat lengths. We estimated nucleosome repeat lengths using distances between MNase midpoint peaks in aggregate plots for the chromosome 12 array region (Figure S12) and genome-wide. The nucleosome repeat length is estimated as the slope of the least squares best fit line. (TIF) [file pgen.1003036.s014.tif]

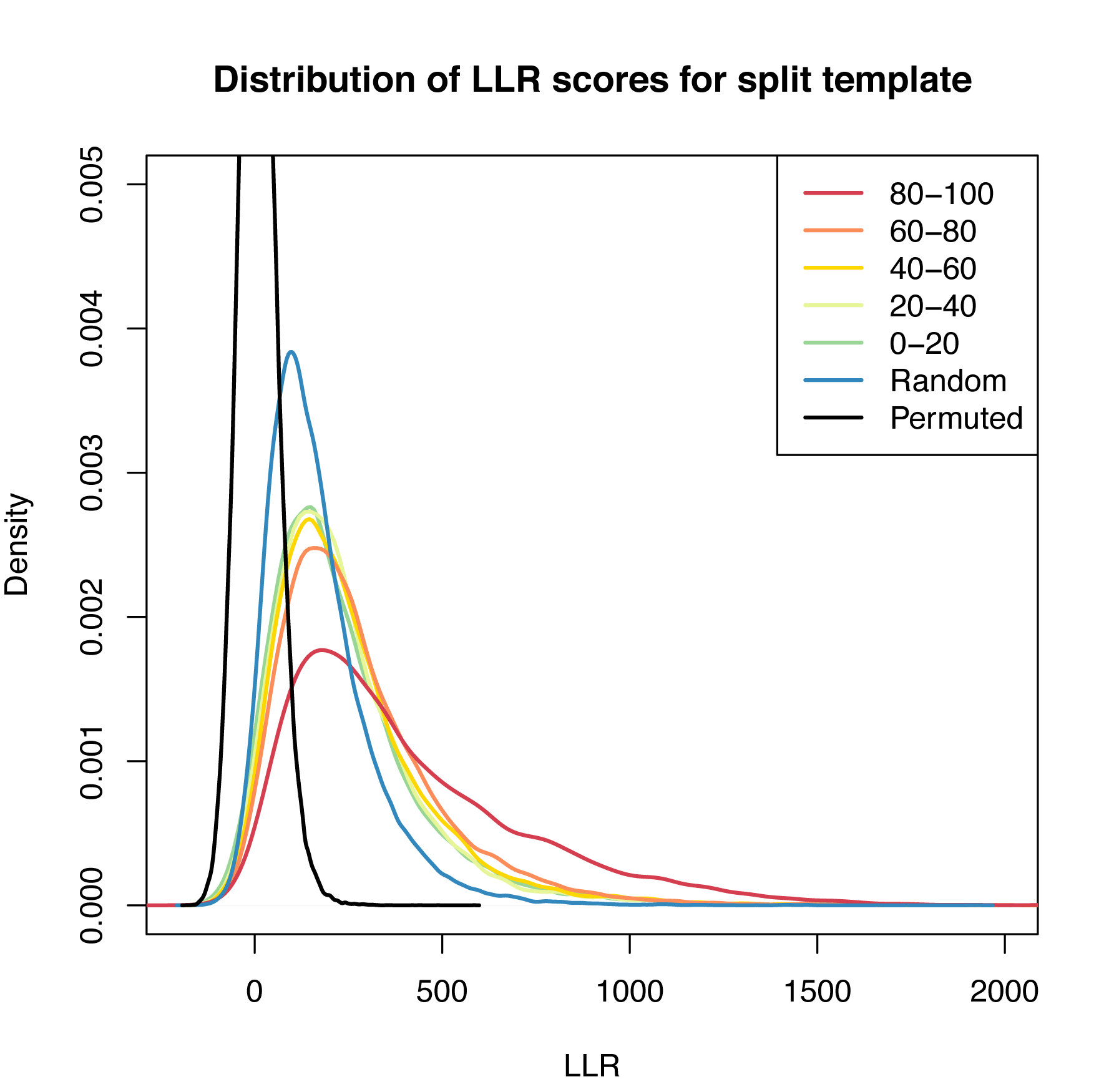

Supplement: Figure S15 — Distribution of nucleosome array log likelihood ratios (LLRs) around ChIP-seq peaks. LLRs around ChIP-seq peaks were calculated using two 1000 bp regions, flanking a central region of variable width, as described in Methods. Separate distributions are plotted for ChIP-seq peaks with different levels of transcription factor occupancy (as described in Figure 5 in the main text). Distributions are also presented for the same regions after permuting the MNase-seq fragment midpoint counts, and for a random sample of genomic regions. (TIF) [file pgen.1003036.s015.tif]

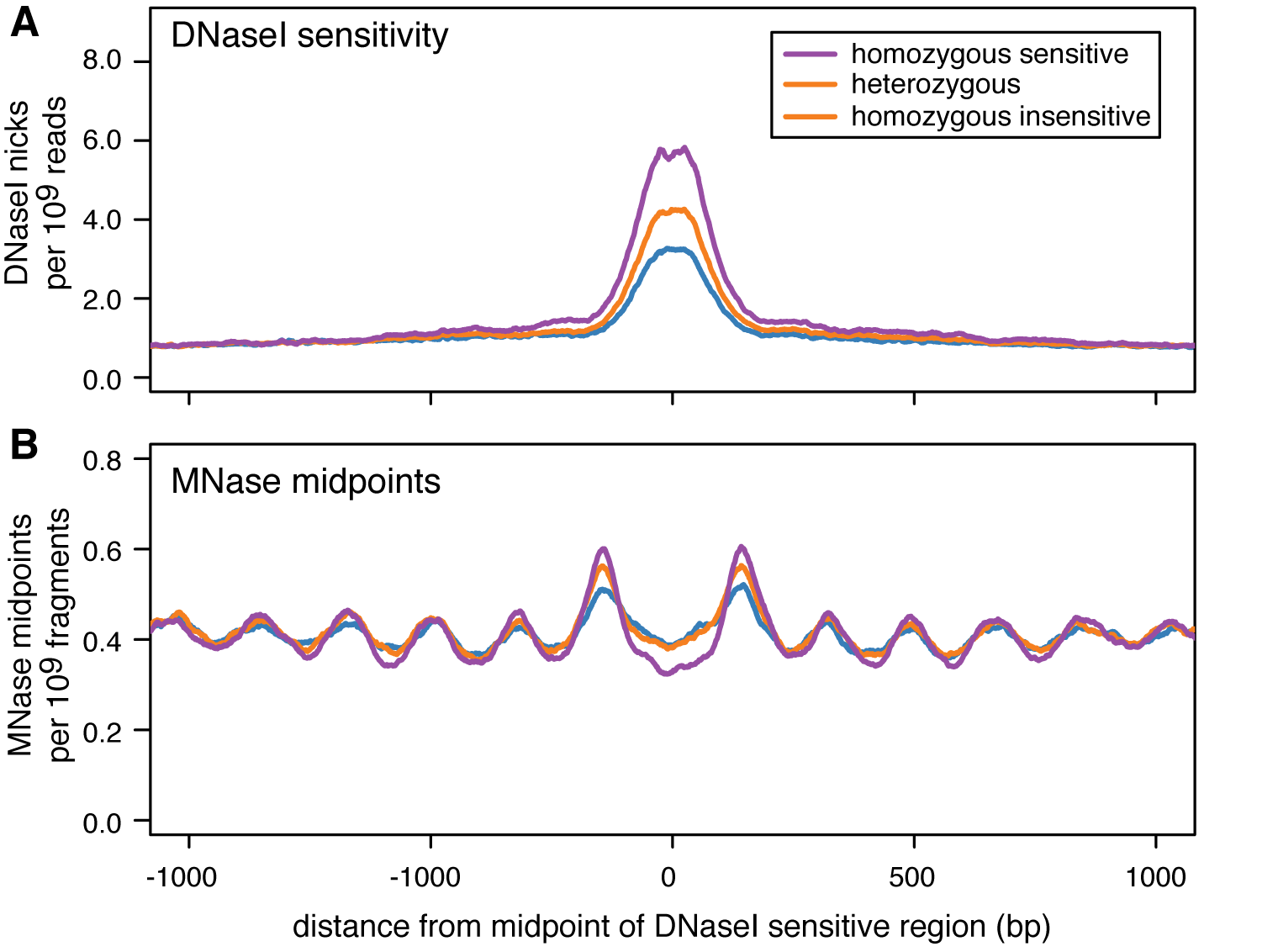

Supplement: Figure S16 — Nucleosome organization in regions with an association between DNase I sensitivity and genotype (dsQTLs). This figure is as described in Figure 6 of the main text, except that data are aggregated across all 4318 filtered dsQTLs, rather than a subset. A. The density of DNase I nicks for different dsQTL genotypes. B. The density of MNase-seq fragment midpoints for different dsQTL genotypes. (TIF) [file pgen.1003036.s016.tif]

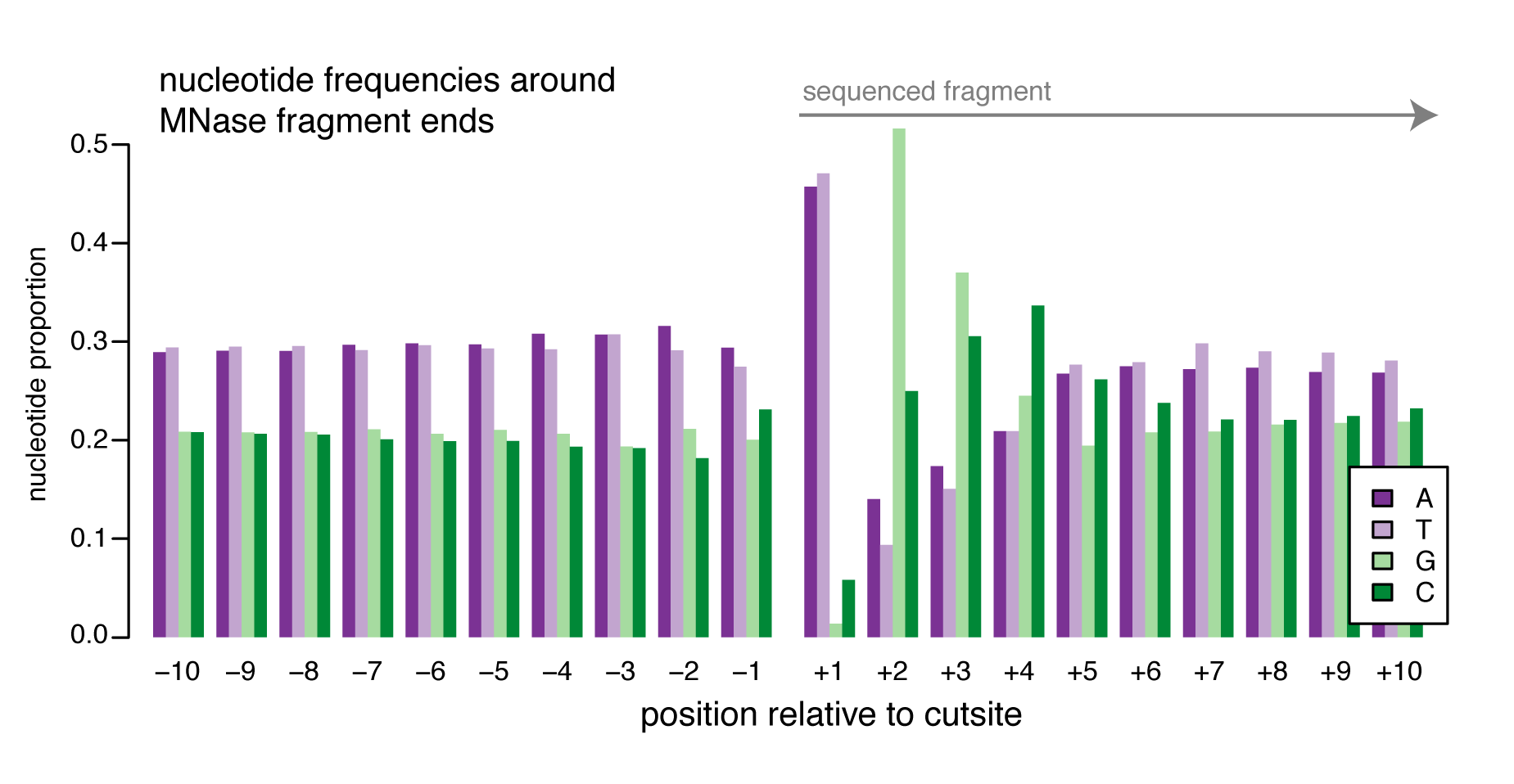

Supplement: Figure S17 — MNase digestion bias. This figure shows the frequency of nucleotides around the ends of MNase-seq fragments that mapped to chromosome 1. K-mers from the first and last four bases of each fragment (+1 to +4) were used to correct for MNase digestion bias, as described in Methods. (TIF) [file pgen.1003036.s017.tif]

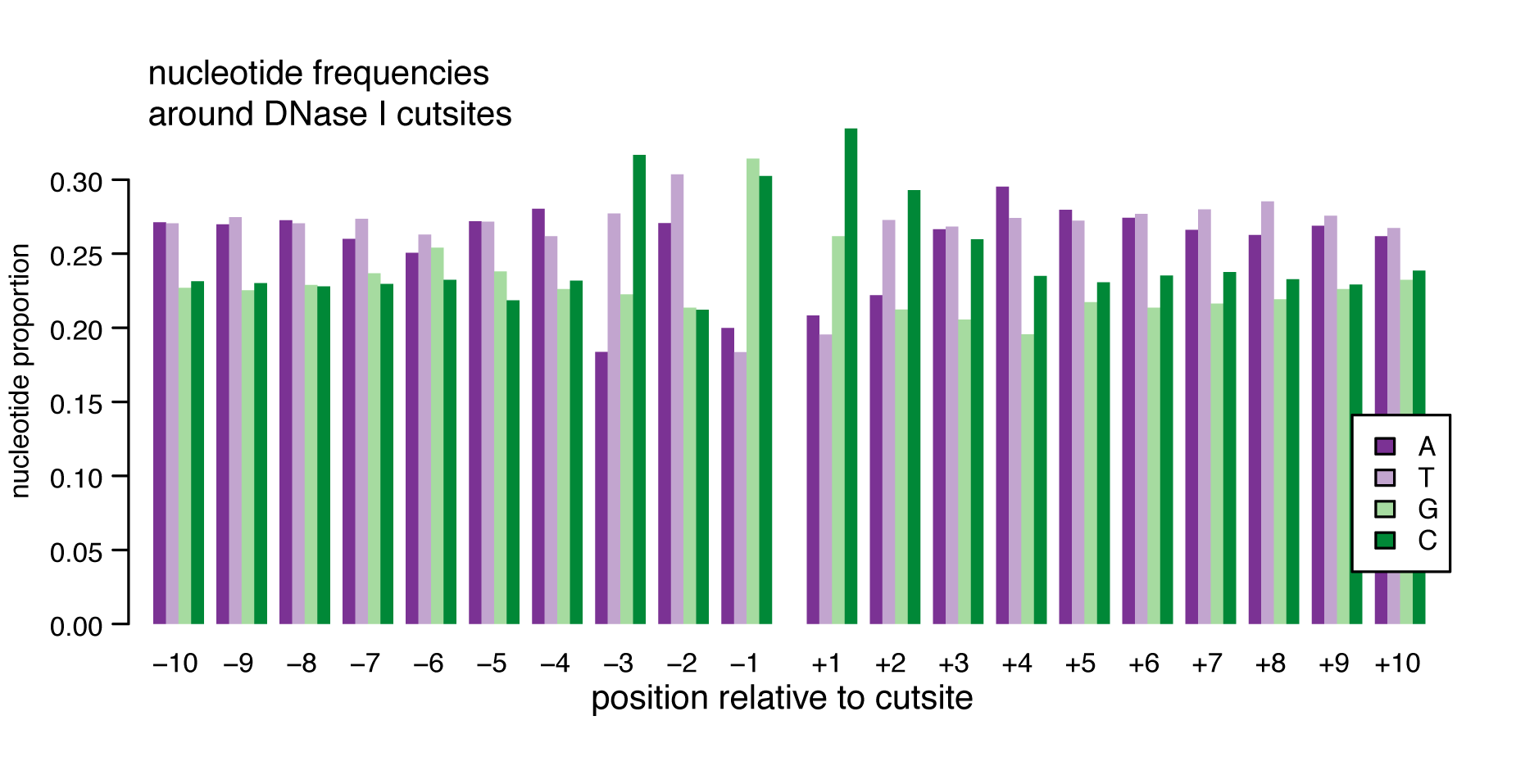

Supplement: Figure S18 — DNaseI cutting bias. This figure shows the frequency of nucleotides around the genomic locations of 3.0 billion DNase I nick sites. K-mers spanning positions −3 to +3 were used to correct for bias in DNase I nicking, as described in Methods. (TIF) [file pgen.1003036.s018.tif]

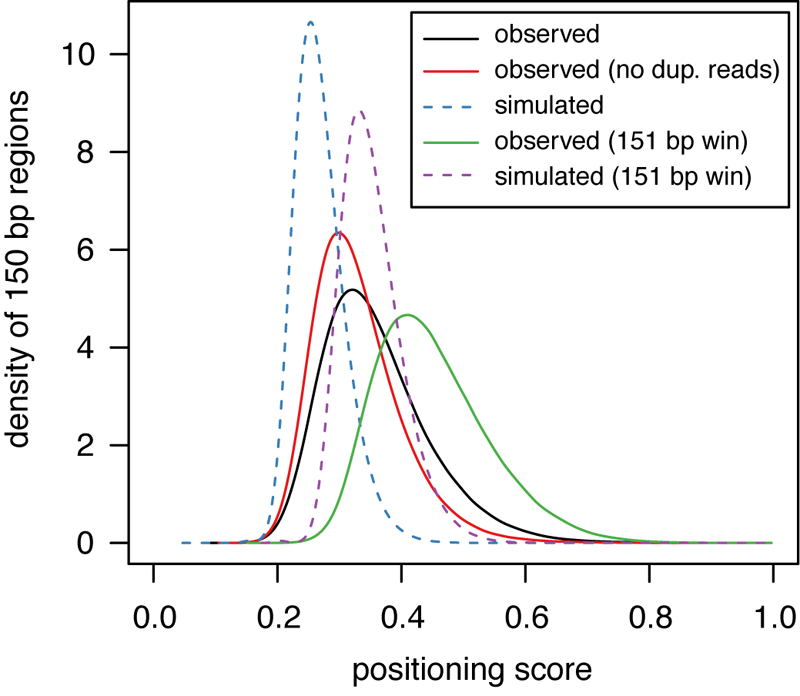

Supplement: Figure S19 — Distributions of nucleosome positioning scores calculated from 150 bp regions. Scores were computed from the same sample of one million regions used in Figure 2, but regions were decreased in size to 150 bp from 200 bp (retaining the same midpoint). The distributions are smoothed using a Gaussian kernel with bandwidth 0.01. For comparison we also calculated scores using 151 bp windows rather than 201 bp windows. (TIF) [file pgen.1003036.s019.tif]
